# The Impact of Free and Nanoencapsulated Banana and Apple Peels Extracts on the Physicochemical, Oxidative Stability, Microbial and Sensory Properties of Whipped Cream

**DOI:** 10.1002/fsn3.70652

**Published:** 2025-07-16

**Authors:** Hamideh Shafahi, Leila Nouri, Seid Mahdi Jafari, Ali Najafi

**Affiliations:** ^1^ Department of Food Science and Technology, Damghan Branch Islamic Azad University Damghan Iran; ^2^ Department of Food Materials and Process Design Engineering Gorgan University of Agricultural Sciences and Natural Resources Gorgan Iran; ^3^ Halal Research Center of IRI, Iran Food and Drug Administration Ministry of Health and Medical Education Tehran Iran

**Keywords:** agricultural wastes, antimicrobials, antioxidants, encapsulation, phenolic compounds

## Abstract

The purpose of this study was to investigate the antioxidant and antimicrobial activity of free and nanoencapsulated banana (BE) and apple (AE) peel extracts on the physicochemical, oxidative stability, antibacterial effect, and sensory characteristics of whipped cream during 28 days of cold storage at 4°C. BE and AE were nanoencapsulated in basil seed gum coating. The particle size, polydispersity index (PDI), and zeta potential of BE and AE‐loaded nanoparticles was 116.5 and 112.2 nm, 0.296 and 0.248, −49.71 and −49.16 mV, respectively. Free extract and apple peel exhibited significantly higher (*p* < 0.05) antioxidant activity than encapsulated extract and banana extract. Encapsulated extract had higher antimicrobial activity against *Staphylococcus aureus* and *E. coli O157:H7*. Free and nanoencapsulated extracts were added to whipped cream formulation at a constant level of 2% w/w. All nanocarriers had a spherical shape without aggregation. pH, apparent viscosity, overrun, drainage, color indexes, peroxide value, thiobarbituric acid, total microbial count, and sensory characteristics of whipped cream were examined. Nanocarriers improved the pH, apparent viscosity, hardness, overrun, oxidative stability, foam stability, and also significantly reduced the acidity, drainage, and microbial load of whipped cream samples during the cold storage period (*p* < 0.05). The antioxidant and antimicrobial activity of nanocarriers at the last days of storage was higher than unencapsulated BE and AE. AE showed a higher preservative effect than BE. All whipped cream samples containing extract had acceptable sensory scores. BE and AE, especially in encapsulated form, have remarkable antioxidant and antimicrobial activities and are able to improve the oxidative stability, antibacterial activity, and physicochemical characteristics of whipped cream. The results of this study suggested the use of 2% w/w of nanoencapsulated peel extract in the formulation of whipped cream.

## Graphical abstract

Apple and banana peel extract contain bioactive phenolic compounds exhibiting antioxidant and antibacterial activity. Free and nanoencapsulated extracts increased the shelf life of whipped cream during cold storage. Nanoencapsulated extract improved the pH, apparent viscosity, hardness, overrun, oxidative stability, and foam stability and also significantly reduced the acidity, drainage, and microbial load of whipped cream samples during the cold storage period. It can be a good alternative to synthetic preservatives. pH, apparent viscosity, overrun, drainage, color indexes, peroxide value, thiobarbituric acid, total microbial count, and sensory characteristics of WC were examined.

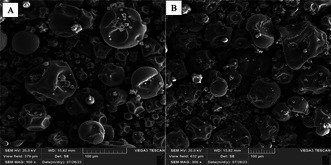

## 1 Introduction

One of the most widely used dairy products in the food industry is whipped cream, which is used in the production of cream pastries, cakes, and desserts (Wang et al. 2023). Stirring the nonhomogenous cream with 35% fat at low temperature in a high‐speed mixer for approximately 2–3 min creates a cream with a stiff texture and increased volume of about 100%–120% (Biglarian et al. 2022). Whipped cream is a complex foam‐like structure based on an emulsion in which fat droplets are partially interconnected and stabilize air bubbles at the air‐water interface (Ghorbani‐HasanSaraei et al. 2019). The high fat content of cream causes lipid oxidation and destructive chemical reactions that generate carcinogenic and toxic compounds in dairy products. This process reduces the nutritional value of the product and limits the shelf life of foods containing fat and oil (Bao and Pignitter 2023). It is also a suitable medium for the growth of microorganisms in various food products that use cream in their formulation and is among the reasons for the spoilage of these products and the poisoning of consumers (Özoğlu and Altuntaş 2019).

Butylated hydroxytoluene (BHT), Butylated hydroxyanisole (BHA), tert‐Butylhydroquinone (TBHQ), and gallate esters are commonly used to increase the shelf life and retard the spoilage reaction rate in dairy products. These synthetic preservatives are inexpensive and readily available, but they have shown high toxicity, carcinogenicity, and mutagenicity in laboratory animals. Today, the consumers tendency towards the use of alternative natural preservatives with antimicrobial and antioxidant functions has increased, and synthetic preservatives are gradually being removed from the list of food preservatives (Mehrabi et al. 2025; Razavi and Kenari 2021; Rodriguez Garcia and Raghavan 2022). Plant essential oils and extracts rich in phenolic compounds can be used to replace synthetic types (Razavi et al. 2024).

A large number of by‐products and wastes are produced during the processing of vegetables and fruits, which are released in the environment without further use and cause environmental problems. Plant wastes are often rich in bioactive compounds such as polyphenols, flavonoids, and carotenoids, and have beneficial effects on human health (Ain et al. 2023; Šovljanski et al. 2023).

Banana (*Musa accuminata*) is an herbaceous plant of the *Musaceae* family. The peel is the most important by‐product of banana, which includes 40% of the fruit weight (Ansari et al. 2023). Banana peel is an excellent source of various bioactive compounds which demonstrate remarkable antimicrobial and antioxidant effects (Hikal et al. 2022). Phenolic compounds of banana peel are in categories of hydroxycinnamic acids, flavanols, flavan‐3‐ols, and catecholamines (Vu et al. 2018). L‐dopa and dopamine have also been found in banana peels, which are known to be significant antioxidants. The yellow color of banana peel is due to the presence of carotenoids such as β‐carotene, α‐carotene, and lutein (Ansari et al. 2023).

Apple (*Malus domastica*) contains high amounts of by‐products such as seeds and peel (Brahmi et al. 2021). It is a rich source of various phenolic compounds and flavonoids, including chlorogenic acid, procyanidins, phlorizin (Radenkovs et al. 2020), chalcones, dihydrochalcones, anthocyanins, flavanols, procyanidin, epicatechin, and catechin, which have strong anti‐radical activity (Bondonno et al. 2017). Besides the various beneficial effects of natural antioxidants on human health, these compounds can delay the oxidation rate of foods and extend the shelf life. However, factors such as temperature, oxygen, humidity, and light have weakening effects on their antioxidant activity and cause destruction (Didar 2020). Encapsulation of plant extracts using biopolymer coatings as wall materials is applied as an effective resolve to increase the stability of bioactive compounds in food systems. This process protects the encapsulated materials against environmental changes, enzymatic and chemical reactions, increases stability against pH changes, contracts ionic and thermal changes, covers the unpleasant taste, odor, and color, and allows controlled release of bioactive substances (Bodbodak et al. 2022).

Various techniques are used to encapsulate bioactive compounds, and emulsification is one of the most common methods (Guía‐García et al. 2022). Plant seed gums, especially basil seed gum (BSG), are one of the most widely used wall materials to encapsulate bioactive compounds (Azarashkan et al. 2022; Jafari et al. 2022). BSG has higher stability, gelling and emulsifying activity, water holding capacity, and film‐forming properties (Guan et al. 2023).

Investigation in different databases demonstrated that the researchers conducted in the field of whipped cream so far have focused on the use of stabilizers to reduce the fat percentage of the cream (Farahmandfar et al. 2019; Wu et al. 2023), and less attention has been paid to improving the shelf life of this product in terms of chemicals and microbes. Therefore, this research was conducted with the aim of investigating the preservation effect of free and nanoencapsulated banana and apple peel extracts in order to extend the shelf life and improve the quality characteristics of whipped cream.

## 2 Materials and Methods

### 2.1 Materials

Ripened and fully mature banana ( *Musa acuminata* ‘Dwarf Cavendish’) and red delicious variety apple were prepared from the local market in Tehran (Iran). The fruits were first washed and their peel was separated by a sharp knife and dried in an oven (Memmert, Germany) at 35°C. Next, the peels were powdered by an electric mill (ParsKhazar, Iran) and sieved with a pore size of 800 μm. BSG was prepared from Caragum Parsian Co. (Tehran, Iran). Standard strains of lyophilized *S. aureus* (PTCC 1015) and *E. coli* (PTCC 1399) were prepared from the microbial collection of the Industrial Research Institute of Iran. Cream with 35% fat was obtained from Iran Dairy Industries Co. (Pegah, Tehran, Iran). The culture medium, gallic acid, quercetin, and analytical grade chemicals used in this research were purchased from Merck Co. (Germany).

### 2.2 Ultrasonic Assisted Extraction of Banana (BE) and Apple (AE) Peel Extracts

BE and AE were obtained using ultrasonic assisted extraction according to the method described by Azizkhani et al. (2023) with slight modifications. In this way, 20 g of each powder was mixed with ethanol: water 50%, and then placed in an ultrasound bath (Bandelin Sonorex, Digitech, Germany) at 40°C, at a frequency of 20 kHz, for 30 min. The obtained extracts were filtered by Whatman No. 1 filter paper, and then their solvents were evaporated using a rotary evaporator (Heidolph, Germany) at 45°C. Extracts were obtained and kept in the freezer (−18°C) until further use.

### 2.3 High Performance Liquid Chromatography (HPLC) of Extracts

The Agilent 1260 series (Wald) was utilized for the HPLC analysis. For the chromatographic separation, an Eclipse C18 column with dimensions of 4.6 × 150 mm and a particle size of 1.8 μm was employed. The mobile phase consisted of water (solvent A) and 0.05% trifluoroacetic acid (TFA) in acetonitrile (solvent B), delivered at a constant flow rate of 0.9 mL/min. The mobile phase was programmed to follow a linear gradient as follows: initially, 82% A at 0 min, decreasing to 80% A over the next 5 min, further reducing to 60% A from 5 to 8 min, maintained at 60% A from 8 to 12 min, increasing back to 82% A from 12 to 15 min, and then maintaining 82% A from 15 to 16 min and continuing until 20 min. The multi‐wavelength detector was set to monitor at 280 nm. Each sample solution was injected with a volume of 5 μL. The column temperature was maintained at 40°C (García et al. 2021).

### 2.4 Nanoencapsulation of BE and AE

To prepare the nanocarriers loaded with BE and AE, 0.05 g BSG was dispersed in deionized water at 30°C. Then, it was cooled and stirred on a magnetic stirrer for 24 h to fully hydrate. After that, extracts (10 mL) were homogenized with Tween 80 (40 mL) and sunflower oil (50 mL) on a magnetic stirrer (15 min at 100 rpm). The final emulsion was homogenized using an Ultraturrax (KND‐1200 UH1‐013, South Korea) for 10 min at 15,000 rpm, and then the coating solution was added to the nanoemulsions in a ratio of 5:1 (Jafari et al. 2022). A spray dryer (Dorsa tech, Iran) was used to dry the nanoemulsion, and the inlet and outlet temperature of the dryer were set at 150°C and 100°C, respectively.

### 2.5 Characterization of Nanoencapsulated BE and AE

The samples were diluted up to 20 times with deionized water. The particle size, PDI, and zeta potential of nanocarriers were measured using a Zetasizer (Malvern, England) device. The encapsulation efficiency (EE) of phenolic compounds in nanocarriers was calculated as a percentage (Mahdi et al. 2020), using the ratio of the encapsulated phenolics to the total phenolic content (TPC) as per Equation (1). EE was determined following the methodology outlined by Mahdi et al. (2020).

(1) $$\mathrm{EE}\;\left(\%\right)=1-\frac{\text{Phenolic compounds}\;\mathrm{on}\;\text{surface}}{\text{Total penolic compounds}\;}\times 100$$

The morphology of nanocarriers was also examined by a scanning electron microscope (SEM) (Tesca‐Vega3, Tescan Co., Czwch Republic) at a voltage of 20 kV and a magnification of ×300.

### 2.6 Total Phenolic/Flavonoid Content (TPC/TFC) and Antioxidant Activity of the Extracts

To measure the TPC, 30 μL of the extract was mixed with 150 μL of Folin‐Ciocaltio reagent (diluted with distilled water in a ratio of 1 to 10) and 120 μL of 7.5% sodium carbonate and was kept in a dark place for 45 min. After color development, the optical absorbance of the mixture against the blank (distilled water) was recorded at 765 nm by a UV–VIS spectrophotometer (UV‐550; Jasco, Japan) (Mehrabi et al. 2025). To measure the TFC, 0.01 g extract was dissolved in ethanol (5 mL) and then 1 mL of the mixture was added to the distilled water and 0.3 mL of sodium nitrite (5%). After 6 min, 2 mL sodium hydroxide solution (1 M) and 6.7 mL distilled water were added to the mixture. The absorbance was recorded at 415 nm using a UV–VIS spectrophotometer. The TPC and TFC were calculated using the standard curve of gallic acid and quercetin (Ranjha et al. 2020). To measure the antioxidant activity of the extracts by the 2,2‐diphenyl‐1‐picrylhydrazyl (DPPH) radical scavenging assay, the extract (50 μL) was mixed with 0.13 mM DPPH solution (1.95 mL) and after the solution was placed at room temperature for 30 min, its optical absorbance was recorded at 517 nm by UV–VIS spectrophotometer (Islam et al. 2021). To measure the antioxidant activity by the ferric reducing antioxidant power (FRAP) assay, first, 0.02 M FeCl_3_.6H_2_O solution in water, 0.01 M 2,4,6‐tripyridyl‐s‐triazine (TPTZ) solution in hydrochloric acid, and 0.3 M phosphate buffer with pH 3.6 were prepared, and then these solutions were mixed together in a ratio of 1:1:10, and the extract (10 μL) was added to them. The mixture was kept for 30 min, and then its optical absorbance was recorded at 593 nm (Esmaeilzadeh Kenari and Razavi 2022).

### 2.7 Antibacterial Activity of the Extracts

Several colonies of microbial suspensions of *S. aureus* (PTCC 1015) and *E. coli* (PTCC 1399) strains were transferred to the nutrient broth culture medium and placed in an incubator at 37°C. Then, 10^6^ CFU/mL of each bacterial suspension equivalent to 0.5 McFarland were prepared in sterile physiological serum, and 100 μL of them was transferred to the surface of plates containing nutrient agar culture medium. 60 μL of extract was transferred to the wells, and the plates were placed in an incubator at 37°C for 24 h. After incubation time, the diameter of the nongrowth zone was measured by a caliper and reported in mm (Fatemi et al. 2024; Jafarpour et al. 2021).

Determination of the minimum inhibitory concentration (MIC) and the minimum bactericidal concentration (MBC) of the extracts was done according to the microdilution method. Different concentrations of extracts were prepared by dimethyl sulfoxide with a dilution of 150 mg/mL. Then, 100 μL of nutrient broth culture was poured into the sterile 96‐well microplates and 100 μL of extract was poured into the wells followed by 100 μL of microbial suspension. After incubation, the turbidity of the wells was investigated and the MIC was considered as the lowest dilution of extract with no turbidity. To determine the MBC values, 5 μL nutrient agar culture was mixed with 100 μL extract and incubated at 37°C for 24 h. The lowest concentration of the extract with no bacterial growth was determined as the MBC (Jafarpour et al. 2021).

### 2.8 Preparation of Whipped Cream

To prepare whipped cream, the fats were removed from the separator and cream was standardized with low‐fat pasteurized milk to 30%. The milk was heated, then 20% sugar and 0.2% milk protein concentrate were added to the milk. Then, whipped cream was dissolved into the milk and they were heated in a hot water bath at 80°C for 10 min. The free and nanoencapsulated extracts were added to whipped cream at a constant level of 2%. A whipped cream containing 100 ppm of BHA antioxidant was considered a positive control. The whipped cream samples were kept at 4°C for 28 days and different tests were performed at days 0, 7, 14, 21, and 28.

### 2.9 Determining Physicochemical Properties of Whipped Creams

The pH of whipped cream was determined by a pH meter (Jenway 3510, England) and their acidity in terms of percentage of lactic acid through titration (Amiri Samani and Naji 2019). The apparent viscosity of whipped cream was measured at 4°C by a rotary viscometer (Brookfield, England) and spindle number 6 (Amiri et al. 2018). To determine the overrun, whipped cream was first stirred for 30 s. The overrun was calculated through Equation (2):

(2) $$\;\text{Overrun}\;\left(\%\right)=\frac{W_{1}-W_{2}}{W_{2}}\times 100$$

where, *W*_1_ and *W*_2_ were the weights of un‐whipped and whipped cream, respectively (Athari et al. 2021):

Drainage test was used to study the stability of whipped cream. The whipped cream was poured into a funnel and after being placed in the refrigerator for 4 h, the separated serum was transferred into a graduated cylinder. The drainage was obtained through the Equation (3) (Li et al. 2020):

(3) $$\text{Drainage}\;\left(\%\right)=\frac{\text{Serum weight}}{\text{Initial cream weight}}\times 100$$

The color indexes of L*, a*, and b* of samples were determined using a colorimeter (Minolta, China) (Amiri et al. 2018). The textural properties of hardness and adhesiveness were investigated at room temperature using a texture analyzer (LLOYD, RS 232, America) with an aluminum cylinder probe (38 mm) and with a probe penetration speed and a penetration distance of 1 mm/s and 30 mm, respectively (Farahmandfar et al. 2017).

### 2.10 Lipid Oxidation

100 g whipped cream was mixed with 400 mL ethanol for 5 h on a shaker, and after the formation of two phases, the oil phase was separated by a strainer. The solvent was evaporated using an oven at 45°C. The peroxide (PV) and the thiobarbituric acid (TBA) value (TBAv) of the oils were measured according to the method described by Jafarpour et al. (2022) and Koohenjani and Lashkari (2022), respectively (Jafarpour et al. 2021; Koohenjani and Lashkari 2022).

### 2.11 Microbial Analysis

The total viable count (TVC) of whipped cream samples was evaluated according to the pour‐plate method. Several consecutive dilutions of samples were prepared using sterile peptone water, and 1 mL of the desired dilution was transferred to the sterile plates. Then, 15–20 mL of Plate Count Agar (PCA) culture was added to each plate, and after 72 h of incubation at 37°C, the number of colonies formed was counted. The results were reported as the logarithm of the number of microorganisms per gram (log CFU/g) (Budhkar et al. 2014).

### 2.12 Sensory Evaluation

Sensory evaluation of whipped cream samples on the day after production was performed by a 5‐point hedonic test by semi‐trained 12 evaluators (six men and six women between the ages of 23 and 40). The samples were provided to the evaluators in a coded form, and the evaluators rated the flavor, texture, odor, color, appearance, and overall acceptability of whipped cream on a 5‐point rating scale, 5 being very good and 1 being very bad (Amiri et al. 2018).

### 2.13 Statistical Analysis of Data

SPSS Ver. 22.0 software and two‐way analysis of variance (ANOVA) were performed to analyze the data obtained from the experiments. The examination of significant differences between samples and day of storage at the probability level of 95% (*p* < 0.05) was done by Duncan's multiple range test. T‐test was used for pairwise comparison between samples. The graphs were drawn using Excel Ver. 2021.

## 3 Results and Discussion

### 3.1 Characteristics and Morphology of Nanocarriers

The characteristics of nanoencapsulated BE and AE are given in Table 1. Particle size is an important and effective factor on the flowability, stability, release, and bioavailability of encapsulated particles. All nanocarriers produced in this research were small in size, and their average particle size was 116.5 and 112.2 nm, respectively. PDI shows the uniformity of the particle size distribution of the particles, and capsules with a PDI < 0.3 are generally ideal (Razavi and Kenari 2021). All nanocarriers had a PDI < 0.3 and therefore had a uniform particle size distribution. Zeta potential indicates the surface charge of the particles, which is related to their stability. All nanocarriers had a negative surface charge, and their zeta potential was obtained as −49.71 and −49.14 mV, respectively. The negative charge of these nanocarriers is due to the anionic nature of plant gums (Tavakoli et al. 2021). Zeta potential values > −30 mV exhibited the good stability of nanocarriers. EE indicates the ability of the wall material to retain bioactive compounds during the encapsulation process. The results showed that nanocarriers had EE > 85% (89.46% and 90.35%, respectively). In the study of Shaygannia et al. (2021), EE of lemon waste extract in BSG was 70.72%, which was lower than the values obtained in the present study. Similar to the results of the present study, Azarashkan et al. (2022) reported that the particle size, PDI, and EE of broccoli sprout extract in BSG were 39.6 nm, 0.279, and 97.96% with negative charge and high stability.

**TABLE 1:** Particle size, PDI, zeta potential, and encapsulation efficiency (EE) of NE‐BE and NE‐AE.

Samples	Mean particle size (nm)	PDI	Zeta potential (mV)	EE (%)
NE‐BE	116.5 ± 2.8^a^	0.296 ± 0.034^a^	−49.71 ± 0.56^a^	89.46 ± 1.07^a^
NE‐AE	112.2 ± 3.6^a^	0.248 ± 0.019^a^	−49.16 ± 0.79^a^	90.35 ± 0.88^a^

*Note:* Values represent mean ± SD. Different letters indicate significant difference among samples (*p* < 0.05). Abbreviations: NE‐AE: nanoencapsulated apple peel extract; NE‐BE: nanoencapsulated banana peel extract.

**FIGURE 1:**
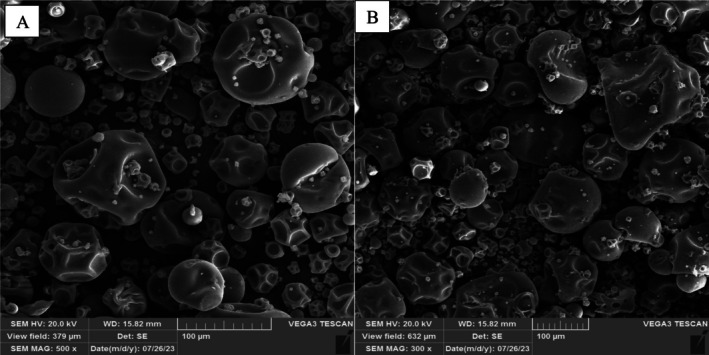
SEM images of (A) NE‐BE and (B) NE‐AE. NE‐AE, nanoencapsulated apple peel extract; NE‐BE, nanoencapsulated banana peel extract.

Figure 1A,B illustrate the morphology of nanocarriers produced in this study, which exhibit a predominantly spherical yet nonuniform shape with surface depressions. Due to the similarity in wall materials, encapsulation techniques, and drying methods employed for both types of nanocarriers, no significant morphological differences were observed between them. Additionally, there was no evidence of particle agglomeration or adhesion, corroborating the high zeta potential and stability of the produced nanocarriers. However, SEM images reveal particles with predominantly spherical morphology, though with noticeable variation in size and surface texture. The spherical shapes are characteristic of spray‐dried particles, where rapid solvent evaporation leads to the formation of nearly spherical droplets that solidify into spherical particles due to surface tension forces aiming to minimize energy. However, deviations from perfect sphericity and the appearance of wrinkled or irregular surfaces may arise due to several contributing factors. These include the potential physicochemical interactions between encapsulated bioactive compounds (e.g., polyphenols) and the wall materials. Furthermore, rapid water removal during spray drying can create internal stresses or partial collapse of the particle shell, leading to surface indentations or irregularities. Such morphological diversity is commonly observed in encapsulation systems involving complex plant extracts and reflects the sensitivity of particle formation to both formulation and processing parameters. This is consistent with findings by Sarabandi et al. (2019), who also observed spherical nanocarriers with uneven, wrinkled surfaces and depressions in eggplant peel extract prepared via spray drying. Similarly, Shaygannia et al. (2021) reported that nanocarriers of lemon waste extract coated with BSG and prepared by spray drying displayed a spherical and slightly nonuniform morphology.

### 3.2 TPC, TFC And Antioxidant Activity of Extracts

The results of TPC, TFC, and antioxidant activity of free and nanoencapsulated extracts are presented in Table 2. The values of TPC (46.53–48.16 mg Gallic Acid Equivalent (GAE)/g), TFC (27.96–28.45 mg Quercetin Equivalent (QE)/g), DPPH (85.92%–88.47%), and FRAP (34.61%–35.54%) of AE were significantly higher than BE (42.92–45.16 mg GAE/g, 19.61–19.82 mg QE/g, 73.43%–76.59% and 28.93%–30.17%, respectively). The free extracts showed a higher antioxidant activity than the nanoencapsulated extracts, which is related to their higher content of phenolic compounds. The surface absorption of some phenolic compounds during the production of capsules, as well as the reaction between the capsule membrane and the extract, can be the reason for reducing the antioxidant activity of the extract after the encapsulating process. Previous research has shown that the encapsulation process leads to only minor reductions in bioactive compounds and antioxidant activity. This indicates that the encapsulation technique effectively maintains the integrity and functional efficacy of these bioactive components. This minimal loss could be related to the effect of the drying process, which can lead to the destruction of some bioactive compounds (Ebrahimian et al. 2024; Fatemi et al. 2024; Gorzin et al. 2024).

**TABLE 2:** TPC, TFC, and antioxidant capacity of free and Nanoencapsulated BE and AE.

Samples	TPC (mg GAE/g)	TFC (mg QE/g)	DPPH (%)	FRAP (%)
Free BE	45.16 ± 0.53^Ba^	19.82 ± 0.19^Bb^	76.59 ± 1.46^Ba^	30.17 ± 0.28^Ba^
NE‐BE	42.92 ± 0.68^Bb^	19.61 ± 0.24^Bb^	73.43 ± 0.86^Bb^	28.93 ± 0.39^Bb^
Free AE	48.16 ± 0.85^Aa^	28.45 ± 0.41^Aa^	88.47 ± 1.38^Aa^	35.54 ± 0.33^Aa^
NE‐AE	46.53 ± 0.39^Ab^	27.96 ± 0.13^Aa^	85.92 ± 1.10^Ab^	34.61 ± 0.17^Ab^

*Note:* Values represent mean ± SD. Different lower‐case letters indicate significant difference among samples with same extract (*p* < 0.05). Different upper‐case letters indicate significant difference among extract with same form (*p* < 0.05). Abbreviations: NE‐AE: nanoencapsulated apple peel extract; NE‐BE: nanoencapsulated banana peel extract.

In the research of Brahmi et al. (2021), TPC and TFC of AE were reported as 19.33 mg GAE/g and 11.61 mg QE/g, respectively, which were lower than the values obtained in the present study. In the study of Kabir et al. (2021), higher TPC (53.80 mg GAE/g), TFC (16.44 mg QE/g) and DPPH (79.07%) were reported for BE compared to the present study. In another study, TPC, TFC, DPPH, and FRAP of ethanolic extract of BE were obtained as 31.46 mg GAE/g, 22.11 mg QE/g, 82.50%, and 25.50%, respectively (Chaudhry et al. 2022). Vieira et al. (2011) also reported the high antioxidant activity of apple peel and attributed this performance to its polyphenol and flavonoid compounds, and these researchers found that the TPC in apple peel was higher than its pulp. The difference in the amounts of bioactive compounds of the extracts in different studies is related to various parameters such as variety, cultivation and harvest conditions, the degree of maturity, processing conditions, extraction methods, and storage conditions (Islam et al. 2021).

### 3.3 Phenolic Composition of Extracts

The results of HPLC analysis in Table 3 revealed 17 major compounds in AE and 17 compounds in BE. Similarly, Kalinowska et al. (2020) measured the phenolic profile of two varieties, Gold Milenium and Papierowka, apple peel extracts obtained using conventional and ultrasound‐assisted methods. They reported the presence of chlorogenic acid, homovanilic acid, epicatechin, rutin, quercetin, phloridzin, and ellagic acid in AE (Kalinowska et al. 2020). In another study conducted by Shehzadi et al. (2020), the presence of polyphenols in different varieties of apples was evaluated, and the presence of catechin, procyanidin, chlorogenic acid, protocatechuic acid, gallic acid, rutin, quercetin, and phloridzin in apple peel extracts was confirmed (Shehzadi et al. 2020), which is in line with the results of the present study. Fotirić Akšić et al. (2022) also reported the foundation of ten phenolic compounds (aesculin, chlorogenic acid, p‐hydroxybenzoic acid, caffeic acid, quercetin, kaempferol, apigenin, phlorizin, phloretin, and baicalein) in apple peel extract (Fotirić Akšić et al. 2022). In terms of banana peel extract, the presence of ellagic acid, gallic acid, ferulic acid, o‐coumaric acid, catechol, salicylic acid, cinnamic acid, rutin, myricetin, and naringenin was reported in previous studies (Aboul‐Enein et al. 2016; Athanasiadis et al. 2023; Behiry et al. 2019).

**TABLE 3:** HPLC phenolic composition of peel extracts.

Compounds	Retention time (min)	(%)	
		AE	BE
Protocatechuic acid	6.19	1.38	4.95
p‐hydroxybenzoic	8.35	0	2.7
Catechin	11.25	0.82	4.45
Quinic acid	14.07	2.6	7.24
Chlorogenic acid	15.23	9.35	1.42
Ellagic acid	17.05	0.92	0.67
Syrngic acid	17.46	0.28	0
Gallic acid	18.62	0.15	2.21
Vanilic acid	20.28	0.11	0.74
Homovanilic acid	23.19	0.05	0.2
Rosmarinic acid	24.45	0	3.48
Ferulic acid	27.36	0.63	4.66
Epicatechin	30.44	2.45	6.87
Procyanidin	31.99	11.25	40.75
Rutin	32.57	1.97	1.88
Kameprol	36.08	3.46	0
Quercitin	40.88	44.35	2.07
Myricetin	43.56	10.22	0.97
Phloridzin	47.29	2.11	4.85
Unknown		7.35	8.25
Total		99.45	98.36

### 3.4 Antibacterial Activity of Extracts

Antibacterial activity of free and nanoencapsulated extracts against two important food pathogenic strains, including *S. aureus* (gram‐positive) and *E. coli* (gram‐negative), was investigated by the well diffusion method. The antibacterial activity of AE against *S. aureus* bacteria was higher than BE, while BE had higher antibacterial activity against *E. coli* than AE (Table 4). The diameter of the inhibitory zone of AEs against *S. aureus* and *E. coli* was 23.58–26.04 and 13.10–14.72 mm, respectively. The diameter of the inhibitory zone of BE against mentioned bacteria strains was 18.13–19.96 and 15.87–17.66 mm, respectively. The MIC and MBC of the extracts also confirmed the inhibitory zone results. The antibacterial activity of plant extracts is related to the presence of different phenolic compounds (Fatemi et al. 2024). These compounds react with important components inside the bacterial cell, including proteins and enzymes, and therefore increase the permeability of the bacterial cell membrane and thereby cause the leakage of intracellular compounds to the outside (Kenari and Razavi 2022). The antibacterial activity of banana peel is related to the presence of flavonoids, alkaloids, terpenoids, tannins, and glycosides (Taweechat et al. 2021). Chaudhry et al. (2022) measured the antibacterial activity of ethanolic extract of banana peel and reported remarkable antibacterial activity against *E. coli* compared to *S. aureus* . The high antimicrobial activity of red AE has been reported in the study conducted by Alpaslan et al. (2021). They stated the presence of phenolic compounds in this extract and its acidity caused the good biological activity of red AE. The remarkable antimicrobial activity of AE has been reported by Van Nguyen and Lee (2023) and Maroufi et al. (2022). The results of this study also indicated that the nanoencapsulation process improved the antibacterial activity of BE and AE against both bacterial strains (*p* < 0.05). The higher antibacterial activity of nanoencapsulated extracts compared to free forms can be attributed to the gradual release of bioactive compounds and their interaction with bacterial cells, which affect the bacterial cell membrane more easily (Azarashkan et al. 2022). In various studies, the size of particles has been identified as a pivotal factor in determining the antimicrobial activity of encapsulated bioactive substances. When the particles are smaller, they exhibit a larger surface area relative to their volume. This increased surface area facilitates greater interaction with microbial cells, leading to improved antimicrobial activity in combating pathogens (Gorzin et al. 2024; Shahidi Noghabi and Molaveisi 2020). Kenari and Razavi (2022) also found that bougainvillea flower extract encapsulated in *Urtica dioca* L. seed gum had higher antimicrobial activity and lower MIC and MBC values against different bacteria compared to the free extract (Kenari and Razavi 2022).

**TABLE 4:** Antibacterial activity of free and nanoencapsulated BE and AE against two major pathogenic bacteria.

Bacteria	Free BE	NE‐BE	Free AE	NE‐AE
**Diameters of inhibitory zone (mm)**				
*S. aureus*	18.13 ± 0.25^Bb^	19.96 ± 0.19^Ba^	23.58 ± 0.31^Ab^	26.04 ± 0.27^Aa^
	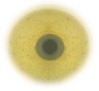	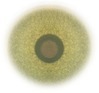	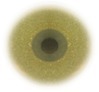	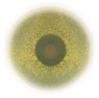
*E. coli*	15.87 ± 0.30^Bb^	17.66 ± 0.23^Ba^	13.10 ± 0.34^Ab^	14.72 ± 0.48^Aa^
	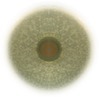	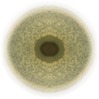	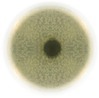	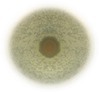
**MIC (mg/mL)**				
*S. aureus*	12.5A^b^	6.25^c^	6.25^c^	6.25^c^
*E. coli*	50.0^b^	25.0^c^	25.0^Ac^	12.50^d^
**MBC (mg/mL)**				
*S. aureus*	25.0^a^	12.5^b^	12.5^b^	6.25^c^
*E. coli*	100^a^	50.0^b^	25.0^a^	12.5^d^

*Note:* Values represent mean ± SD. Different lower‐case letters indicate significant difference among samples with same extract (*P* < 0.05). Different upper‐case letters indicate significant difference among extract with same form (*p* < 0.05). Abbreviations: NE‐AE: nanoencapsulated apple peel extract; NE‐BE: nanoencapsulated banana peel extract.

### 3.5 pH And Acidity

**FIGURE 2:**
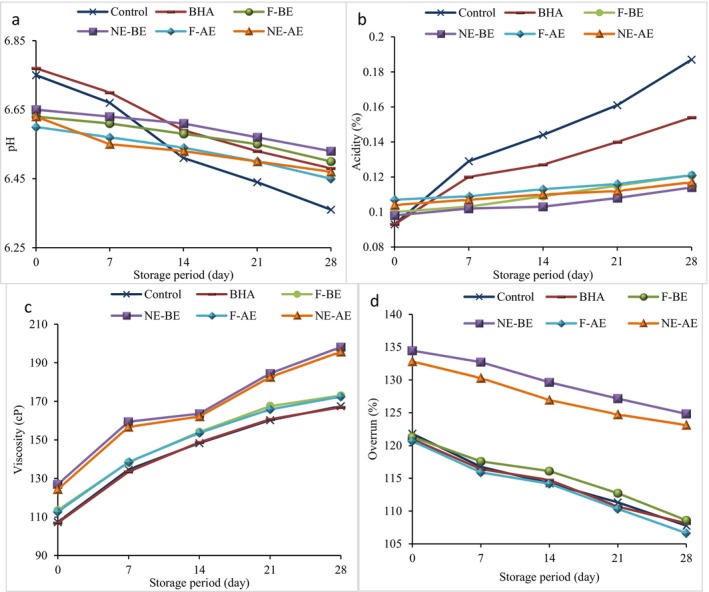
Changes in pH (a), acidity (b), apparent viscosity values (c), and overrun (d) of whipped cream samples during cold storage. F‐AE, free apple peel extract; F‐BE, free banana peel extract; NE‐AE, nanoencapsulated apple peel extract; NE‐BE, nanoencapsulated banana peel extract..

The changes in pH and acidity values of whipped cream during 28 days of storage at 4°C are shown in Figure 2. At the first time of storage, the control sample and whipped cream containing BHA antioxidant had the highest pH (6.75 and 6.77, respectively) and the lowest acidity (similarly; 0.093%). The pH of whipped cream decreased significantly (*p* < 0.05) by adding different forms of BE and AE to the presence of phenolic acids in these extracts and reached 6.60–6.65 (Figure 2a), and their acidity values increased (*p* < 0.05) and reached 0.098–0.107% (Figure 2b). From the first day of storage up to the end, a significant decrease in pH and an increase in the acidity values of different whipped cream treatments were observed (*p* < 0.05). The lower antimicrobial activity in the control sample and in whipped cream containing synthetic antioxidant exhibited the fastest changes in pH and acidity. The reduction of pH and acidity changes in whipped cream containing AE and BE is due to their antimicrobial activity. On day 28, the control sample had the lowest pH (6.36) and the highest acidity (0.187%), and whipped cream containing NE‐BE showed the highest pH (6.53) and the lowest acidity (0.114%). Overall, the type of additives used in the formulation and the growth of microorganisms are two major and effective factors on the pH and acidity of dairy products (Kavak and Akdeniz 2019). In the research of Brahmi et al. (2021), Ahmad et al. (2020) and El‐Messery et al. (2019), it was also observed that the pH of yogurt decreased and its acidity increased when AE was added to the formulation of the product. The decrease in pH and increase in acidity of dairy desserts during storage was also reported in the research of Okur (2023).

### 3.6 Apparent Viscosity

Apparent viscosity is one of the major parameters in dairy products, especially whipped cream, which has a significant impact on the technological aspects of the product. The viscosity of whipped cream is also effective on consumer acceptance and its organoleptic characteristics (Athari et al. 2021). The results of the apparent viscosity of whipped cream samples during 28 days of storage (Figure 2c) showed that at the beginning of the storage, the addition of NE‐BE and NE‐AE to the formulation caused a significant increase in the apparent viscosity compared to other treatments (*p* < 0.05). This increase was due to the use of BSG for encapsulating the extracts. Hydrocolloids can improve the apparent viscosity of the product by absorbing water in their structure. Also, with the addition of nanocarriers, the apparent viscosity of the product increased by increasing the solid content. Demirkol and Tarakci (2018) reported that the incorporation of extracts rich in phenolic compounds can rearrange the gel network in dairy products through the reaction between phenolic compounds and milk casein, thus improving the stability of the product network (Demirkol and Tarakci 2018). During storage time, higher apparent viscosity of whipped cream samples containing nanocarriers compared to the others was observed. On the first day of storage time, the apparent viscosity was in the range of 106.9–126.9 cP and reached 166.9–198.1 cP over time, which is due to the increase in the solid content (*p* < 0.05) of whipped cream samples. Other researchers also reported that the addition of banana peel powder in enriched yogurts (Kabir et al. 2021), gums in low‐fat whipped cream (Lakzadeh and Nasr Esfahani 2021) and nanoencapsulated grape seed in yogurt (Yadav et al. 2018) increments the apparent viscosity.

### 3.7 Overrun

Overrun indicates the air in whipped cream. During the aeration process, air is placed into the liquid mixture and forms the foam, which is a physical phenomenon. Due to the transformation of the liquid into a foam, the volume increases, and this increase in volume compared to the initial volume is called overrun (Lakzadeh and Nasr Esfahani 2021). Whipped cream samples that have a higher overrun often demonstrate higher texture hardness and foam stability (Gaba and Anand 2023). The results of investigating the overrun of whipped cream samples enriched with BE and AE are shown in Figure 2d. At the beginning of storage, the addition of NE‐BE and NE‐AE to the whipped cream formulation increments the overrun compared to the control sample (*p* < 0.05). The overrun of whipped cream samples on day 0 was in the range of 120.67%–134.48%. During storage time, the highest overrun was related to whipped cream enriched with nanocarriers, and it decreased to 106.63%–124.83% over time. Viscosity has a significant effect on increasing the overrun of whipped cream samples, which is related to the placement of air bubbles in the cream formulation (Seo and Yoo 2022). The results of the present study showed that the addition of nanocarriers, by increasing the viscosity, could improve the overrun of whipped cream. The type of extract had no significant effect on the overrun of whipped cream samples. During storage, the overrun of all whipped cream samples gradually decreased (*p* < 0.05), which is probably related to the increase in viscosity and the hardening of the texture of whipped cream. The relationship between increasing viscosity and improving the overrun of whipped cream has also been observed in the study of Athari et al. (2021). They stated that stabilizers such as gums are able to limit the movement of air bubbles and thus reduce the rate of air bubble aggregation and thereby improve the overrun of whipped cream. Gaba and Anand (2023) also reported that the addition of probiotic capsules increased the overrun of whipped cream, which is attributed to the use of gums for the production of capsules.

### 3.8 Drainage of Whipped Creams

**FIGURE 3:**
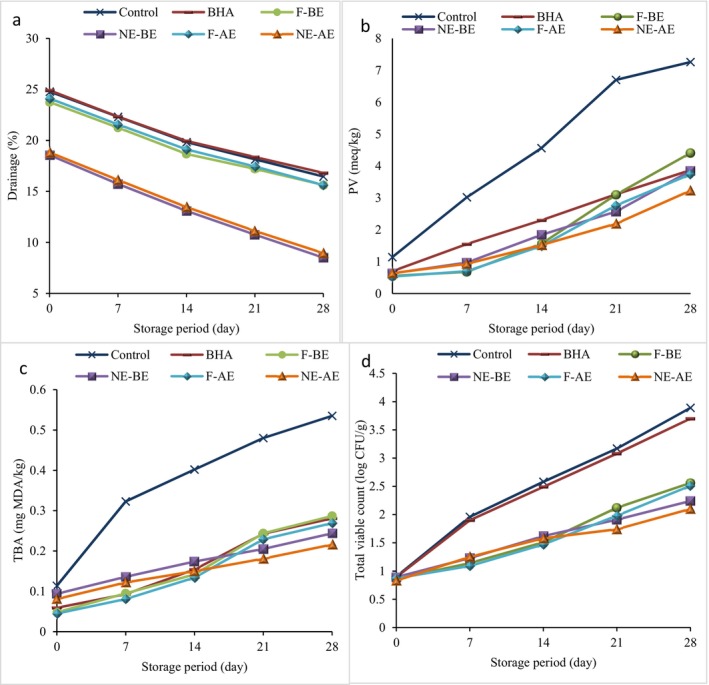
Changes in drainage (a), PV (b) TBA values (c), and total viable count (d) of whipped cream treatments during the cold storage. F‐AE, free apple peel extract; F‐BE, free banana peel extract; NE‐AE, nanoencapsulated apple peel extract; NE‐BE, nanoencapsulated banana peel extract.

Drainage indicates the amount of serum removed from whipped cream. Overall, cream is an oil in water emulsion, and therefore its drainage showed the breakdown of the emulsion (Lakzadeh and Nasr Esfahani 2021). Figure 3a shows the changes in the drainage values of whipped cream samples during 28 days of storage at 4°C. At the beginning of the storage, the highest drainage was observed in the control sample (24.78%) and whipped cream containing synthetic antioxidant (24.91%). The addition of AE and BE to the whipped cream formulation caused a significant decrease in the drainage percentage (*p* < 0.05). The drainage values of whipped cream samples enriched with the extracts on day 0 of storage were in the range of 18.56%–24.11%. During the storage, the drainage decreased (*p* < 0.05) and reached 8.51%–16.81%, which is related to the absorption of water and its trapping in the stabilizer network. Whipped cream has the desired quality and the least drainage (Lakzadeh and Nasr Esfahani 2021) which indicates the higher stability of whipped cream foam. In general, drainage occurs as a result of the difference between the pressure of air bubbles inside and outside. The higher intensity of air bubbles pressure compared to the internal liquid pressure caused the entrance of air bubbles into the bubble spaces, and thus drainage or serum separation occurs (Gaba and Anand 2023). Drainage has a closely negative correlation with the apparent viscosity of dairy products, and products with higher viscosity have less drainage. Therefore, the reason for the significant decrease in whipped cream drainage due to the incorporation of NE‐BE and NE‐AE is related to the increase in viscosity. BSG, which was used to encapsulate the extracts, by absorbing water and keeping it in its structure, improves whipped cream viscosity and reduces drainage. In line with these results, Ahmed et al. (2022) showed that the addition of apple and pomegranate peel powders dried by freeze dryer decreased the drainage of yogurts. In the research of Farahmandfar et al. (2017) and Biglarian et al. (2022), the use of BSG at low concentration improved the foam stability of whipped cream. In general, the researchers stated that plant seed gums are able to improve foam stability and reduce serum separation, which is related to creating a viscoelastic layer around the fat globules, reducing partial aggregation, and increasing viscosity (Rezvani et al. 2020).

### 3.9 Color of Whipped Creams

The color of the products has an effect on their acceptance by the consumer and therefore it is one of the important characteristics of foods. Color is also very effective in evaluating the quality of food and its freshness (Sukkwai et al. 2018). The results of evaluating the color of whipped cream by calorimeter are given in Table 5. At the beginning of the storage, the addition of free and nanoencapsulated BE and AE to whipped cream formulation due to the color of these extracts and the presence of anthocyanins and carotenoids caused a significant decrease in the L* and an increase in a* and b* compared to the control sample (*p* < 0.05). The L*, a*, and b* of whipped cream treatments on the first day were in the range of 61.23–72.10, 0.23–8.89, and 9.14–17.83, respectively. In all the days studied in this research, the lowest value of L* and the highest value of a* was related to whipped cream containing free AE, while whipped cream containing free BE had the highest value of b*. As expected, the changes in color indexes in whipped cream containing nanocarriers were significantly less than the free form of the extracts, because one of the prominent features of the encapsulation process is the covering of the organoleptic properties of the core or central substances. During the storage, there was no significant change in the L*, a*, and b* of whipped cream. In the research of El‐Messery et al. (2019), with the increase in the level of microencapsulated AP in the yogurt formulation, the color of the samples gradually became darker, redder, and yellower. Yadav et al. (2018) reported that the brightness of yogurts containing free grape extract was significantly lower than the control sample, but despite the lower L* of yogurts containing nanoencapsulated extract, these products were not significantly different from the control in terms of brightness. In the research of Kabir et al. (2021), a decrease in the L* and b* and an increase in the a* of yogurts were observed due to the incorporation of BE.

**TABLE 5:** Comparing the color indexes of whipped cream treatments during the cold storage.

	Treatments	0	7	14	21	28
L*	Control	72.10 ± 0.97^Aa^	71.35 ± 0.68^Aa^	70.82 ± 0.83^Aa^	70.48 ± 1.05^Aa^	70.06 ± 0.94^Aa^
	BHA	71.85 ± 0.73^Aa^	71.41 ± 0.84^Aa^	71.19 ± 1.10^Aa^	70.93 ± 0.89^Aa^	70.55 ± 1.03^Aa^
	BE	63.46 ± 0.79^Ac^	63.43 ± 0.96^Ac^	63.28 ± 0.75^Ac^	62.87 ± 0.92^Ac^	62.69 ± 0.70^Ac^
	NE‐BE	65.94 ± 0.67^Ab^	65.87 ± 0.81^Ab^	65.83 ± 0.88^Ab^	65.56 ± 0.95^Ab^	65.30 ± 0.54^Ab^
	AE	61.23 ± 0.94^Ad^	61.12 ± 0.73^Ad^	60.94 ± 0.62^Ad^	60.81 ± 0.77^Ad^	60.57 ± 0.98^Ad^
	NE‐AE	72.10 ± 0.97^Aa^	71.35 ± 0.68^Aa^	70.82 ± 0.83^Aa^	70.48 ± 1.05^Aa^	70.06 ± 0.94^Aa^
a*	Control	0.28 ± 0.07^A,e^	0.31 ± 0.03^A,e^	0.29 ± 0.09^A,e^	0.35 ± 0.07^A,e^	0.37 ± 0.06^A,e^
	BHA	0.23 ± 0.05^A,e^	0.27 ± 0.07^A,e^	0.24 ± 0.03^A,e^	0.27 ± 0.03^A,e^	0.31 ± 0.09^A,e^
	BE	2.47 ± 0.06^A,c^	2.45 ± 0.04^A,c^	2.48 ± 0.05^A,c^	2.47 ± 0.04^A,c^	2.43 ± 0.04^A,c^
	NE‐BE	0.86 ± 0.03^A,d^	0.91 ± 0.06^A,d^	0.92 ± 0.05^A,d^	0.86 ± 0.02^A,d^	0.89 ± 0.03^A,d^
	AE	8.89 ± 0.04^A,a^	8.93 ± 0.03^A,a^	8.91 ± 0.01^A,a^	8.94 ± 0.02^A,a^	8.94 ± 0.03^A,a^
	NE‐AE	0.28 ± 0.07^A,e^	0.31 ± 0.03^A,e^	0.29 ± 0.09^A,e^	0.35 ± 0.07^A,e^	0.37 ± 0.06^A,e^
b*	Control	8.81 ± 0.34^A,e^	8.85 ± 0.21^Ae^	8.86 ± 0.30^A,e^	8.91 ± 0.16^A,e^	8.97 ± 0.27^A,e^
	BHA	9.14 ± 0.19^A,e^	9.23 ± 0.33^Ae^	9.17 ± 0.24^A,e^	9.24 ± 0.29^A,e^	9.21 ± 0.21^A,e^
	BE	17.83 ± 0.24^A,a^	17.84 ± 0.28^Aa^	17.91 ± 0.17^A,a^	17.97 ± 0.23^A,a^	17.94 ± 0.35^A,a^
	NE‐BE	13.05 ± 0.27 ^A,b^	13.11 ± 0.21^Ab^	13.10 ± 0.36^A,b^	13.19 ± 0.30^A,b^	13.08 ± 0.14^A,b^
	AE	11.69 ± 0.33 ^A,c^	11.84 ± 0.18^Ac^	11.93 ± 0.22^A,c^	11.87 ± 0.17^A,c^	11.95 ± 0.23^A,c^
	NE‐AE	8.81 ± 0.34 ^A,e^	8.85 ± 0.21^Ae^	8.86 ± 0.30^A,e^	8.91 ± 0.16^A,e^	8.97 ± 0.27^A,e^

*Note:* Values represent mean ± SD. Lower‐ and upper‐case letters indicate significant difference among treatments and storage period (*p* < 0.05), respectively. Abbreviations: AE, apple peel extract; BE, banana peel extract; NE‐AE, nanoencapsulated apple peel extract; NE‐BE, nanoencapsulated banana peel extract.

### 3.10 Texture of Whipped Creams

Texture is an important and effective feature on edible quality in food processing conditions. Hardness shows the force used for a specific change in the product, and in the case of whipped cream, it is related to the strength of gel. The hardness of whipped cream should be such that, after aeration, the air bubbles in whipped cream can be maintained to a suitable amount and the created foam can remain stable (Athari et al. 2021). The adhesiveness parameter is used to express the flow capacity or the liquid state of the compounds. In general, it is the work required to overcome the attraction force between the surfaces of the food and the surface of the materials that are in contact with them (Farahmandfar et al. 2017). Hardness and adhesiveness are two important textural parameters for whipped cream. The results of evaluating the texture of whipped cream are given in Table 6. At the beginning of the storage, by adding NE‐BE and NE‐AE to whipped cream formulation, a significant increase in the hardness of whipped cream was observed (*p* < 0.05), which was related to the increase in solid content and the increase in viscosity. However, with the addition of extracts, there was no significant change in the adhesiveness of whipped cream (*p* > 0.05). The hardness and adhesiveness values of whipped cream on the first day were in the range of 75.37–78.13 g and 29.50–29.81 mJ, respectively. At all time periods, whipped cream containing NE‐BE and NE‐AE had the highest hardness. From the first day of storage to the last day, an increase in the hardness of different whipped cream treatments was observed (*p* < 0.05), and on the last day, the hardness of whipped cream reached 79.27–84.83 g. Despite the slight increase in the adhesiveness of whipped cream over time, these changes were not significant. Farahmandfar et al. (2017) demonstrated that BSG contributed to an increase in the hardness of whipped cream, without significantly affecting its adhesiveness, which aligns with the findings of the present study. This enhancement in hardness, and consequently the improved stability of whipped cream upon the addition of BSG, has also been corroborated by other researchers (Biglarian et al. 2022, 2021).

**TABLE 6:** Comparing the textural parameters of whipped cream treatments during the cold storage.

	Treatments	0	7	14	21	28
Hardness (g)	Control	75.84 ± 0.56^Db^	76.42 ± 0.35^CD,b^	77.19 ± 0.62^bc,b^	77.84 ± 0.46^B,b^	79.35 ± 0.39^A,b^
	BHA	75.49 ± 0.69^Db^	76.51 ± 0.46^CD,b^	77.38 ± 0.49^bc,b^	78.10 ± 0.63^B,b^	79.57 ± 0.51^A,b^
	BE	76.31 ± 0.62^Cb^	76.58 ± 0.40^C,b^	77.24 ± 0.55^bc,b^	78.21 ± 0.42^B,b^	79.64 ± 0.29^A,b^
	NE‐BE	78.13 ± 0.48^Da^	80.56 ± 0.52^C,a^	81.96 ± 0.63^B,a^	83.15 ± 0.61^B,a^	84.83 ± 0.57^A,a^
	AE	75.37 ± 0.64^Db^	76.30 ± 0.58^CD,b^	77.15 ± 0.41 ^bc,b^	77.86 ± 0.56^B,b^	79.27 ± 0.45^A,b^
	NE‐AE	77.94 ± 0.43^Ea^	80.14 ± 0.65^D,a^	81.39 ± 0.53^C,a^	82.85 ± 0.34^B,a^	84.26 ± 0.39^A,a^
Adhesiveness (mJ)	Control	29.64 ± 0.37^A,a^	29.70 ± 0.26^A,a^	29.81 ± 0.31 ^A,a^	29.86 ± 0.18^A,a^	30.11 ± 0.35^A,a^
	BHA	29.57 ± 0.30^A,a^	29.63 ± 0.39^A,a^	29.82 ± 0.22^A,a^	29.94 ± 0.33^A,a^	30.05 ± 0.24^A,a^
	BE	29.68 ± 0.17^A,a^	29.81 ± 0.24^A,a^	29.76 ± 0.15^A,a^	29.84 ± 0.36^A,a^	30.19 ± 0.37^A,a^
	NE‐BE	29.81 ± 0.26^A,a^	29.97 ± 0.18^A,a^	30.01 ± 0.34^A,a^	30.11 ± 0.29^A,a^	30.14 ± 0.18^A,a^
	AE	29.50 ± 0.22^A,a^	29.61 ± 0.35^A,a^	29.64 ± 0.27^A,a^	29.75 ± 0.14^A,a^	29.91 ± 0.33^A,a^
	NE‐AE	29.50 ± 0.22^A,a^	29.61 ± 0.35^A,a^	29.64 ± 0.27^A,a^	29.75 ± 0.14^A,a^	29.91 ± 0.33^A,a^

*Note:* Values represent mean ± SD. Lower‐ and upper‐ case letters indicate significant difference among treatments and storage period (*p* < 0.05), respectively. Abbreviations: AE, apple peel extract; BE, banana peel extract; NE‐AE, nanoencapsulated apple peel extract; NE‐BE, nanoencapsulated banana peel extract.

### 3.11 Lipid Oxidation of Whipped Creams

#### 3.11.1 Peroxide Value (PV)

Lipid oxidation is a detrimental process that leads to the degradation of nutritional value, the loss of bioactive compounds, the formation of harmful and toxic substances, and negatively impacts the sensory properties of foods containing oils or fats. The PV serves as an oxidative marker to assess the formation of hydroperoxides during lipid oxidation. Hydroperoxides, being primary oxidation products, are unstable and decompose into secondary oxidation products over time (Razavi et al. 2021). Figure 3b illustrates the changes in PV across various whipped cream samples over a 28‐day storage. Initially, the addition of extracts in both free and encapsulated forms significantly reduced the PV of whipped cream due to the potent antioxidant activity of these extracts (*p* < 0.05). The control sample exhibited the highest PV, measured at 1.14 meq/kg. In contrast, the incorporation of different forms of extracts led to a significant reduction (*p* < 0.05) in the rate of hydroperoxide formation during storage, with PV ranging from 0.53 to 0.64 meq/kg. This reduction is attributed to the high antioxidant capacity of the compounds present in the extracts. Throughout the storage, a significant increase in PV was observed in all whipped cream samples (*p* < 0.05). However, due to the protective effects of the plant bioactive compounds, the nanoencapsulation process effectively preserved the antioxidant activity of the extracts (Abdolshah et al. 2025; Khalili et al. 2024). Consequently, by the end of the storage, the antioxidant efficacy of nanocarriers in whipped cream was markedly higher than that of the free extracts. Consistently, Shaygannia et al. (2021) demonstrated that encapsulated lemon waste extract with BSG exhibited superior antioxidant activity compared to the free extract, leading to a more substantial reduction in the PV of mayonnaise. Polyphenols, known for their hydrogen‐donating ability and high radical‐scavenging capacity, exhibit significant antioxidant properties and are recognized as potent natural antioxidants (Hadidi et al. 2022). Kabir et al. (2021) found that banana peel extract (BE) significantly mitigated the formation of hydroperoxides in enriched yogurts relative to the control, attributing its antioxidant efficacy to the presence of phenolic acids (e.g., hydroxycinnamic acids), flavonoids (e.g., dopamine, quercetin, L‐dopa, and catecholamines), and anthocyanins (Rebello et al. 2014). Similarly, Ahmad et al. (2020) reported enhanced antioxidant activity in yogurt upon the incorporation of apple peel extract (AE).

#### 3.11.2 Thiobarbituric Acid Value

TBA is one of the most commonly used indicators for assessing secondary oxidation products. The variations in TBAv of whipped cream samples over 28 days of storage at 4°C are depicted in Figure 3c. Initially, the control sample exhibited the highest TBAv = 0.114 mg MDA/kg. The addition of extracts significantly reduced (*p* < 0.05) TBAv in whipped cream samples, bringing them to a range of 0.045–0.094 mg MDA/kg. Over the storage period, a significant increase in TBAv was observed in all samples (*p* < 0.05), with the control sample, which lacked preservatives, showing the largest rise. By the end of the storage period, the highest TBAv was recorded in the control sample (0.535 mg MDA/kg), while the whipped cream sample containing nanoencapsulated apple extract (NE‐AE) exhibited the lowest TBAv (0.216 mg MDA/kg). Overall, whipped cream samples with nanoencapsulated extracts maintained lower TBAv than those with free extracts by the end of the storage period. This enhanced antioxidant activity in delaying lipid oxidation in whipped cream parallels findings in other studies. Anwar et al. (2023) reported an improvement in the antioxidant capacity of yogurt enriched with banana extract (BE). Similarly, Khalid et al. (2021) observed a significant reduction in secondary oxidation products in mayonnaise with the addition of apple extract (AE). The results of this study clearly demonstrate the potent antioxidant activity of the extracts in inhibiting lipid oxidation in whipped cream, with AE showing a superior effect compared to the synthetic antioxidant BHA. Despite BE's notable antioxidant capacity, its nanoencapsulated form outperformed the free extract. On the final day of storage, the enhanced preservation and stability of phenolic and bioactive compounds in nanocarriers resulted in higher antioxidant activity.

### 3.12 Total Viable Count of Whipped Creams

The changes in TVC of whipped cream samples throughout the storage are presented in Figure 3d. Initially, no significant differences (*p* > 0.05) were observed in the TVC of the various samples, which ranged from 0.83 to 0.90 log CFU/g. Over the storage, a significant increase in microbial load was noted in all samples (*p* < 0.05), with the control sample exhibiting the most rapid increase in TVC due to the absence of antimicrobial additives. By the end of the storage, the control sample had the highest TVC, followed by the sample containing a synthetic antioxidant. In contrast, whipped cream with encapsulated extracts exhibited the lowest TVC levels, measuring 1.10 and 2.24 log CFU/g, respectively. The antimicrobial activity of banana and apple peel extracts is attributed to the presence of phenolic compounds, which are also present in BSG. These phenolic compounds induce physiological changes in bacterial cell membranes, leading to bacterial death (Riaz et al. 2018). Furthermore, as discussed in section 3.4, the nanoencapsulation process enhances the antimicrobial efficacy of these extracts by facilitating the gradual release of antimicrobial agents. Consequently, whipped cream enriched with nanoencapsulated extracts demonstrated lower microbial loads compared to those containing free extracts throughout the storage, corroborating their antimicrobial effectiveness under in vitro conditions. Consistent with these findings, Anwar et al. (2023) reported that banana extract, rich in bioactive compounds, effectively reduced microbial load and extended the shelf life of yogurt. Similarly, Shaygannia et al. (2021) observed that during 30 days of storage, the TVC of mayonnaise samples increased; however, the addition of lemon waste extract, particularly in its encapsulated form, significantly lowered the microbial load of the mayonnaise samples.

### 3.13 Sensory Evaluation of Whipped Creams

**FIGURE 4:**
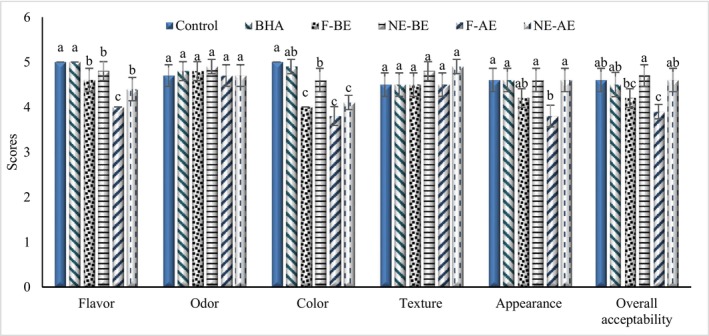
Comparing the sensory characteristics of whipped cream treatments. F‐AE, free apple peel extract; F‐BE, free banana peel extract; NE‐AE, nanoencapsulated apple peel extract; NE‐BE, nanoencapsulated banana peel extract.

The sensory evaluation scores of whipped cream samples, taken one day postproduction, are illustrated in Figure 4. The incorporation of BE and AE extracts led to a decrease in flavor, color, appearance, and overall acceptability of whipped cream samples. However, the impact of the nanoencapsulated extracts was less pronounced compared to their free form counterparts, likely due to the encapsulation process mitigating the effects of the extracts on sensory properties. Notably, the texture and odor of whipped cream remained statistically unaffected by the addition of the extracts. Panelists reported slight changes in flavor, color, and appearance for whipped cream enriched with free extracts. Despite these changes, all samples, particularly those containing nanoencapsulated extracts, were deemed acceptable with favorable sensory characteristics. Consistent with these findings, Anwar et al. (2023) observed an enhancement in yogurt flavor upon the addition of BE, while Kabir et al. (2021) reported no adverse impact on the sensory acceptance of yogurt with BE supplementation. Ahmad et al. (2020) also noted that adding apple peel extract improved the sensory attributes of yogurt, including flavor, color, and texture, which aligns with the results of this study.

## 4 Conclusion

The growing consumer preference for healthier foods with natural and functional additives has spurred increased research into the utilization of plant extracts in food products. The findings of this study reveal that the incorporation of encapsulated extracts into whipped cream formulations significantly increased viscosity and hardness, leading to reduced drainage and enhanced overrun and foam stability. Both BE and AE extracts exhibited notable antioxidant and antimicrobial activities in their free and nanoencapsulated forms. Notably, the antioxidant and antimicrobial efficacy of the nanoencapsulated extracts in whipped cream samples was superior to that of the free extracts towards the end of the storage. Overall, AE was more effective than BE in delaying lipid oxidation and reducing microbial load in whipped cream. Although the addition of these extracts induced some modifications in the sensory properties of whipped cream, all samples remained acceptable and received favorable evaluations. Ultimately, the results suggest that the incorporation of nanoencapsulated AE can effectively extend the shelf life and preserve the quality of whipped cream during refrigerated storage.

## Author Contributions

**Hamideh Shafahi:** conceptualization – equal, investigation – equal, software‐equal, writing – review and editing‐equal. **Leila Nouri:** funding acquisition – equal, supervision – equal, validation – equal, writing – review and editing – equal. **Seid Mahdi Jafari:** data curation – equal, formal analysis – equal, visualization – equal, writing – original draft – equal. **Ali Najafi:** methodology – equal, project administration – equal, resources – equal, writing – review and editing – equal.

## Ethics Statement

All ethical issues, especially sensory evaluation, have been verified by the Islamic Azad University of Damghan Branch (IR. IAU.1402.1234). Signed informed consents were collected from all the participants of this study.

## Conflicts of Interest

The authors declare no conflicts of interest.

## Supporting information


Figure S1.

Table S5‐S9.

Data S1.

## Data Availability

Data will be made available on request.

## References

[fsn370652-bib-0001] Abdolshah, M. , A. Najafi , R. Razavi , and S. Marboutian . 2025. “Effect of Curcumin Loaded in Nanoemulsion Basil Seed Gum Coating on the Reduction of Acrylamide and Oil Absorption in Fried Potato Strips.” Food Research International 200: 115467. 39779107 10.1016/j.foodres.2024.115467

[fsn370652-bib-0002] Aboul‐Enein, A. M. , Z. A. Salama , A. A. Gaafar , H. F. Aly , F. Abou‐Elella , and H. Ahmed . 2016. “Identification of Phenolic Compounds From Banana Peel (Musa Paradaisica L.) as Antioxidant and Antimicrobial Agents.” Journal of Chemical and Pharmaceutical Research 8, no. 4: 46–55.

[fsn370652-bib-0003] Ahmad, I. , A. Khalique , M. Q. Shahid , et al. 2020. “Studying the Influence of Apple Peel Polyphenol Extract Fortification on the Characteristics of Probiotic Yoghurt.” Plants 9, no. 1: 77. 10.3390/plants9010077. 31936135 PMC7020425

[fsn370652-bib-0004] Ahmed, M. , A. Ali , A. Sarfraz , Q. Hong , and H. Boran . 2022. “Effect of Freeze‐Drying on Apple Pomace and Pomegranate Peel Powders Used as a Source of Bioactive Ingredients for the Development of Functional Yogurt.” Journal of Food Quality 2022: 1–9. 10.1155/2022/3327401.

[fsn370652-bib-0005] Ain, H. B. U. , T. Tufail , S. Bashir , et al. 2023. “Nutritional Importance and Industrial Uses of Pomegranate Peel: A Critical Review.” Food Science & Nutrition 11, no. 6: 2589–2598. 10.1002/fsn3.3320. 37324891 PMC10261788

[fsn370652-bib-0006] Alpaslan, D. , T. Ersen Dudu , and N. Aktas . 2021. “Synthesis of Smart Food Packaging From Poly (Gelatin‐Co‐Dimethyl Acrylamide)/citric Acid‐Red Apple Peel Extract.” Soft Materials 19, no. 1: 64–77. 10.1080/1539445X.2020.1765802.

[fsn370652-bib-0007] Amiri, A. , A. Mousakhani‐Ganjeh , S. Torbati , G. Ghaffarinejhad , and R. Esmaeilzadeh Kenari . 2018. “Impact of High‐Intensity Ultrasound Duration and Intensity on the Structural Properties of Whipped Cream.” International Dairy Journal 78: 152–158. 10.1016/j.idairyj.2017.12.002.

[fsn370652-bib-0008] Amiri Samani, S. , and M. H. Naji . 2019. “Effect of Homogenizer Pressure and Temperature on Physicochemical, Oxidative Stability, Viscosity, Droplet Size, and Sensory Properties of Sesame Vegetable Cream.” Food Science & Nutrition 7, no. 3: 899–906. 10.1002/fsn3.680. 30918632 PMC6418465

[fsn370652-bib-0009] Ansari, N. A. I. M. , N. Z. Ramly , N. Huda‐Faujan , and N. Arifin . 2023. “Nutritional Content and Bioactive Compounds of Banana Peel and Its Potential Utilization: A Review.” Malaysian Journal of Science, Health & Technology (MJoSHT) 9, no. 1: 74–86. 10.33102/MJOSHT.V9I1.313.

[fsn370652-bib-0010] Anwar, S. , S. Javid , Q. A. Syed , et al. 2023. “Screening and Quantification of Non‐Thermally Extracted Antioxidants and Phytochemicals From Banana Peels via LC‐ESI‐QTOF‐MS/MS and Their Functionality in Spoonable Plain‐Yogurt.” Journal of Food Measurement and Characterization 18: 1133–1143. 10.1007/s11694-023-02215-w.

[fsn370652-bib-0011] Athanasiadis, V. , T. Chatzimitakos , M. Mantiniotou , D. Kalompatsios , E. Bozinou , and S. I. Lalas . 2023. “Investigation of the Polyphenol Recovery of Overripe Banana Peel Extract Utilizing Cloud Point Extraction.” Eng 4, no. 4: 3026–3038.

[fsn370652-bib-0012] Athari, B. , A. Nasirpour , S. Saeidy , and A. Esehaghbeygi . 2021. “Physicochemical Properties of Whipped Cream Stabilized With Electrohydrodynamic Modified Cellulose.” Journal of Food Processing and Preservation 45, no. 9: e15688.

[fsn370652-bib-0013] Azarashkan, Z. , S. Farahani , A. Abedinia , et al. 2022. “Co‐Encapsulation of Broccoli Sprout Extract Nanoliposomes Into Basil Seed Gum: Effects on in Vitro Antioxidant, Antibacterial and Anti‐Listeria Activities in Ricotta Cheese.” International Journal of Food Microbiology 376: 109761. 10.1016/j.ijfoodmicro.2022.109761. 35661556

[fsn370652-bib-0014] Azizkhani, M. , S. Kavosi , and R. Partovi . 2023. “Improving the Quality of the Chicken Fillet Using Chitosan, Gelatin, and Starch Coatings Incorporated With Bitter Orange Peel Extract During Refrigeration.” Food Science & Nutrition 11, no. 8: 4700–4712. 10.1002/fsn3.3432. 37576027 PMC10420770

[fsn370652-bib-0015] Bao, Y. , and M. Pignitter . 2023. “Mechanisms of Lipid Oxidation in Water‐In‐Oil Emulsions and Oxidomics‐Guided Discovery of Targeted Protective Approaches.” Comprehensive Reviews in Food Science and Food Safety 22, no. 4: 2678–2705. 10.1111/1541-4337.13158. 37097053 PMC10962568

[fsn370652-bib-0016] Behiry, S. I. , M. K. Okla , S. A. Alamri , et al. 2019. “Antifungal and Antibacterial Activities of Musa Paradisiaca L. Peel Extract: HPLC Analysis of Phenolic and Flavonoid Contents.” PRO 7, no. 4: 215.

[fsn370652-bib-0017] Biglarian, N. , A. Atashzar , A. Rafe , and S. A. Shahidi . 2022. “Effect of Rice Bran Protein and Modified Basil Seed Gum on Physicochemical Properties and Stability of Confectionery Cream.” Journal of Food Science and Technology (Iran) 18, no. 121: 13–24. 10.52547/fsct.18.121.2.

[fsn370652-bib-0018] Biglarian, N. , A. Rafe , and S. A. Shahidi . 2021. “Effect of Basil Seed Gum and κ‐Carrageenan on the Rheological, Textural, and Structural Properties of Whipped Cream.” Journal of the Science of Food and Agriculture 101, no. 14: 5851–5860. 10.1002/jsfa.11237. 33788968

[fsn370652-bib-0019] Bodbodak, S. , M. Nejatian , A. P. Ghandehari Yazdi , et al. 2022. “Improving the Thermal Stability of Natural Bioactive Ingredients via Encapsulation Technology.” Critical Reviews in Food Science and Nutrition 64, no. 10: 2824–2846. 10.1080/10408398.2022.2127145. 36178297

[fsn370652-bib-0020] Bondonno, N. P. , C. P. Bondonno , N. C. Ward , J. M. Hodgson , and K. D. Croft . 2017. “The Cardiovascular Health Benefits of Apples: Whole Fruit vs. Isolated Compounds.” Trends in Food Science & Technology 69: 243–256. 10.1016/j.tifs.2017.04.012.

[fsn370652-bib-0021] Brahmi, F. , F. Merchiche , S. Mokhtari , et al. 2021. “Optimization of Some Extraction Parameters of Phenolic Content From Apple Peels and Grape Seeds and Enrichment of Yogurt by Their Powders: A Comparative Study.” Journal of Food Processing and Preservation 45, no. 2: e15126. 10.1111/jfpp.15126.

[fsn370652-bib-0022] Budhkar, Y. , S. Bankar , and R. Singhal . 2014. “Microbiology of Cream and Butter.” Encyclopedia of Food Microbiology 1: 728–737. 10.1016/B978-0-12-384730-0.00221-4.

[fsn370652-bib-0023] Chaudhry, F. , M. L. Ahmad , Z. Hayat , et al. 2022. “Extraction and Evaluation of the Antimicrobial Activity of Polyphenols From Banana Peels Employing Different Extraction Techniques.” Separations 9, no. 7: 165. 10.3390/separations9070165.

[fsn370652-bib-0024] Demirkol, M. , and Z. Tarakci . 2018. “Effect of Grape ( *Vitis labrusca* L.) Pomace Dried by Different Methods on Physicochemical, Microbiological and Bioactive Properties of Yoghurt.” LWT 97: 770–777. 10.1016/j.lwt.2018.07.058.

[fsn370652-bib-0025] Didar, Z. 2020. “Characterization of White Chocolate Enriched With Free or Encapsulated Pomegranate Extract.” Journal of Nutrition, Fasting and Health 8, no. 4: 302–309. 10.22038/jnfh.2020.50603.1281.

[fsn370652-bib-0026] Ebrahimian, P. , A. Najafi , and A. Abedinia . 2024. “Effect of Nanoencapsulated Pistachio Green Hull Extract in the Carboxymethyl Cellulose and Soy Protein Isolate Edible Coatings on Shelf‐Life Quality of Fresh Pistachio.” Journal of Food Processing and Preservation 2024, no. 1: 5524814.

[fsn370652-bib-0027] El‐Messery, T. M. , M. M. El‐Said , E. Demircan , and B. Ozçelik . 2019. “Microencapsulation of Natural Polyphenolic Compounds Extracted From Apple Peel and Its Application in Yoghurt.” Acta Scientiarum Polonorum. Technologia Alimentaria 18, no. 1: 25–34. 10.17306/J.AFS.2019.0597. 30927749

[fsn370652-bib-0028] Esmaeilzadeh Kenari, R. , and R. Razavi . 2022. “Phenolic Profile and Antioxidant Activity of Free/Bound Phenolic Compounds of Sesame and Properties of Encapsulated Nanoparticles in Different Wall Materials.” Food Science & Nutrition 10: 525–535. 35154689 10.1002/fsn3.2712 PMC8825734

[fsn370652-bib-0029] Farahmandfar, R. , M. Asnaashari , M. R. Salahi , and T. Khosravi Rad . 2017. “Effects of Basil Seed Gum, Cress Seed Gum and Quince Seed Gum on the Physical, Textural and Rheological Properties of Whipped Cream.” International Journal of Biological Macromolecules 98: 820–828. 10.1016/j.ijbiomac.2017.02.046. 28212934

[fsn370652-bib-0030] Farahmandfar, R. , M. Asnaashari , A. Taheri , and T. K. Rad . 2019. “Flow Behavior, Viscoelastic, Textural and Foaming Characterization of Whipped Cream: Influence of Lallemantia Royleana Seed, Salvia Macrosiphon Seed and Carrageenan Gums.” International Journal of Biological Macromolecules 121: 609–615. 10.1016/j.ijbiomac.2018.09.163. 30268748

[fsn370652-bib-0031] Fatemi, A. , A. Najafi , R. Razavi , and S. Jafarzadeh . 2024. “Characterizing the Antioxidant and Antifungal Properties of Nano‐Encapsulated Pistachio Hull Extract in Fenugreek Seed Gum to Maintain the Quality and Safety of Fresh Pistachio.” Food Science & Nutrition 12: 5561–5571. 39139972 10.1002/fsn3.4209 PMC11317734

[fsn370652-bib-0032] Fotirić Akšić, M. , D. Dabić Zagorac , U. Gašić , T. Tosti , M. Natić , and M. Meland . 2022. “Analysis of Apple Fruit (Malus× Domestica Borkh.) Quality Attributes Obtained From Organic and Integrated Production Systems.” Sustainability 14, no. 9: 5300.

[fsn370652-bib-0033] Gaba, K. , and S. Anand . 2023. “Potential of Incorporating a Functional Probiotic Encapsulant in Whipped Cream.” Fermentation 9, no. 11: 928. 10.3390/fermentation9110928.

[fsn370652-bib-0034] García, P. , C. Fredes , I. Cea , et al. 2021. “Recovery of Bioactive Compounds From Pomegranate (*Punica Granatum* L.) Peel Using Pressurized Liquid Extraction.” Food 10, no. 2: 203. 10.3390/foods10020203 PMC7909278 33498325

[fsn370652-bib-0035] Ghorbani‐HasanSaraei, A. , A. Rafe , S.‐A. Shahidi , and A. Atashzar . 2019. “Microstructure and Chemorheological Behavior of Whipped Cream as Affected by Rice Bran Protein Addition.” Food Science & Nutrition 7, no. 2: 875–881. 10.1002/fsn3.939. 30847166 PMC6392820

[fsn370652-bib-0036] Gorzin, M. , M. Saeidi , S. Javidi , E.‐K. Seow , and A. Abedinia . 2024. “Nanoencapsulation of Oliveria Decumbens Vent./Basil Essential Oils Into Gum Arabic/Maltodextrin: Improved in Vitro Bioaccessibility and Minced Beef Meat Safety.” International Journal of Biological Macromolecules 270: 132288. 38735604 10.1016/j.ijbiomac.2024.132288

[fsn370652-bib-0037] Guan, L. , Y. Ma , F. Yu , et al. 2023. “The Recent Progress in the Research of Extraction and Functional Applications of Basil Seed Gum.” Heliyon 9: e19302. 10.1016/j.heliyon.2023.e19302. 37662748 PMC10472252

[fsn370652-bib-0038] Guía‐García, J. L. , A. V. Charles‐Rodríguez , M. H. Reyes‐Valdés , et al. 2022. “Micro and Nanoencapsulation of Bioactive Compounds for Agri‐Food Applications: A Review.” Industrial Crops and Products 186: 115198. 10.1016/j.indcrop.2022.115198.

[fsn370652-bib-0039] Hadidi, M. , J. C. Orellana‐Palacios , F. Aghababaei , D. J. Gonzalez‐Serrano , A. Moreno , and J. M. Lorenzo . 2022. “Plant By‐Product Antioxidants: Control of Protein‐Lipid Oxidation in Meat and Meat Products.” LWT 169: 114003. 10.1016/j.lwt.2022.114003.

[fsn370652-bib-0040] Hikal, W. M. , S.‐A. Ahl , A. Hussein , et al. 2022. “Banana Peels: A Waste Treasure for Human Being.” Evidence‐based Complementary and Alternative Medicine 2022: 7616452. 10.1155/2022/7616452. 35600962 PMC9122687

[fsn370652-bib-0041] Islam, M. R. , A. R. Haque , M. R. Kabir , M. M. Hasan , K. J. Khushe , and S. K. Hasan . 2021. “Fruit By‐Products: The Potential Natural Sources of Antioxidants and α‐Glucosidase Inhibitors.” Journal of Food Science and Technology 58: 1715–1726. 10.1007/s13197-020-04681-2. 33897010 PMC8021657

[fsn370652-bib-0042] Jafari, S. Z. , S. Jafarian , M. Hojjati , and L. Najafian . 2022. “Evaluation of Antioxidant Activity of Nano‐ and Microencapsulated Rosemary ( *Rosmarinus officinalis* L.) Leaves Extract in Cress ( *Lepidium sativum* ) and Basil ( *Ocimum basilicum* ) Seed Gums for Enhancing Oxidative Stability of Sunflower Oil.” Food Science & Nutrition 10, no. 6: 2111–2119. 10.1002/fsn3.2827. 35702297 PMC9179134

[fsn370652-bib-0043] Jafarpour, D. , S. M. B. Hashemi , and A. Ghaedi . 2021. “Study the Antibacterial Properties of Different Parts of Saffron Extract and Their Application in Cream.” Journal of Food Science and Technology 18, no. 115: 339–349. 10.52547/fsct.18.115.27.

[fsn370652-bib-0044] Kabir, M. R. , M. M. Hasan , M. R. Islam , A. R. Haque , and S. M. K. Hasan . 2021. “Formulation of Yogurt With Banana Peel Extracts to Enhance Storability and Bioactive Properties.” Journal of Food Processing and Preservation 45, no. 3: e15191. 10.1111/jfpp.15191.

[fsn370652-bib-0045] Kalinowska, M. , K. Gryko , A. M. Wróblewska , A. Jabłońska‐Trypuć , and D. Karpowicz . 2020. “Phenolic Content, Chemical Composition and Anti−/Pro‐Oxidant Activity of Gold Milenium and Papierowka Apple Peel Extracts.” Scientific Reports 10, no. 1: 14951. 32917912 10.1038/s41598-020-71351-w PMC7486935

[fsn370652-bib-0046] Kavak, D. D. , and B. Akdeniz . 2019. “Physicochemical Characteristics and Antioxidant Capacity of Traditional Yogurt Fortified With Grape (*Vitis vinifera* L.) Seed Extract at Different Levels.” Kocatepe Veterinary Journal 12, no. 4: 398‐395. 10.30607/kvj.596784.

[fsn370652-bib-0047] Kenari, R. E. , and R. Razavi . 2022. “Encapsulation of Bougainvillea ( *Bougainvillea spectabilis* ) Flower Extract in *Urtica dioica* L. Seed Gum: Characterization, Antioxidant/Antimicrobial Properties, and in Vitro Digestion.” Food Science & Nutrition 10, no. 10: 3436–3443. 36249979 10.1002/fsn3.2944 PMC9548349

[fsn370652-bib-0048] Khalid, M. U. , M. A. Shabbir , S. Mustafa , et al. 2021. “Effect of Apple Peel as an Antioxidant on the Quality Characteristics and Oxidative Stability of Mayonnaise.” Applied Food Research 1, no. 2: 100023. 10.1016/j.afres.2021.100023.

[fsn370652-bib-0049] Khalili, M. , A. Najafi , and R. Razavi . 2024. “Preservative Activity of Free and Nano‐Encapsulated Pomegranate Peel Extract Obtained Using Cold Plasma and Ultrasound‐Assisted Method in Increasing the Shelf Life of Thigh Mutton Mince.” Applied Food Research 5: 100668.

[fsn370652-bib-0050] Koohenjani, D. K. , and H. Lashkari . 2022. “Effects of Double Emulsion Encapsulated Iron on the Properties of Fortified Cream.” LWT 161: 113296. 10.1016/j.lwt.2022.113296.

[fsn370652-bib-0051] Lakzadeh, L. , and S. Nasr Esfahani . 2021. “Application of the Xanthan Gum, Carboxymethyl Cellulose and Whey Protein Concentrate in the Formulation and Improvement of Low Fat Whipped Cream Properties.” FSCT 18, no. 113: 273–287. 10.52547/fsct.18.113.273.

[fsn370652-bib-0052] Li, Y. , Y. Li , D. Yuan , Y. Wang , M. Li , and L. Zhang . 2020. “The Effect of Caseins on the Stability and Whipping Properties of Recombined Dairy Creams.” International Dairy Journal 105: 104658. 10.1016/j.idairyj.2020.104658.

[fsn370652-bib-0053] Mahdi, A. A. , J. K. Mohammed , W. Al‐Ansi , et al. 2020. “Microencapsulation of Fingered Citron Extract With Gum Arabic, Modified Starch, Whey Protein, and Maltodextrin Using Spray Drying.” International Journal of Biological Macromolecules 152: 1125–1134. 10.1016/j.ijbiomac.2019.10.201. 31751737

[fsn370652-bib-0054] Maroufi, L. Y. , N. Shahabi , M. d. Ghanbarzadeh , and M. Ghorbani . 2022. “Development of Antimicrobial Active Food Packaging Film Based on Gelatin/Dialdehyde Quince Seed Gum Incorporated With Apple Peel Polyphenols.” Food and Bioprocess Technology 15, no. 3: 693–705. 10.1007/s11947-022-02774-8.

[fsn370652-bib-0055] Mehrabi, M. , M. Amiri , R. Razavi , A. Najafi , and A. Hajian‐Tilaki . 2025. “Influence of Varied Processing Methods on the Antioxidant Capacity, Antibacterial Activity, and Inviolability of Iranian Black, Oolong, and Green Leafy Teas.” Food Chemistry 464, no. 2: 141793. 39486218 10.1016/j.foodchem.2024.141793

[fsn370652-bib-0056] Okur, Ö. D. 2023. “Utilization of Natural Plant Sources in a Traditional Dairy Dessert, Muhallebi.” Cogent Food & Agriculture 9, no. 1: 2200601. 10.1080/23311932.2023.2200601.

[fsn370652-bib-0057] Özoğlu, Ö. , and E. G. Altuntaş . 2019. “Efficiency of a Herbal Liquid Extract Mixture for the Prevention of Salmonella Growth in Whipped Cream.” Natural and Engineering Sciences 4, no. 1: 65–75.

[fsn370652-bib-0058] Radenkovs, V. , T. Püssa , K. Juhnevica‐Radenkova , et al. 2020. “Wild Apple (Malus spp.) by‐Products as a Source of Phenolic Compounds and Vitamin C for Food Applications.” Food Bioscience 38: 100744. 10.1016/j.fbio.2020.100744.

[fsn370652-bib-0059] Ranjha, M. M. A. N. , S. Amjad , S. Ashraf , et al. 2020. “Extraction of Polyphenols From Apple and Pomegranate Peels Employing Different Extraction Techniques for the Development of Functional Date Bars.” International Journal of Fruit Science 20, no. sup3: S1201–S1221. 10.1080/15538362.2020.1782804.

[fsn370652-bib-0060] Razavi, R. , and R. E. Kenari . 2021. “Antioxidant Evaluation of *Fumaria parviflora* L. Extract Loaded Nanocapsules Obtained by Green Extraction Methods in Oxidative Stability of Sunflower Oil.” Journal of Food Measurement and Characterization 15, no. 3: 2448–2457.

[fsn370652-bib-0061] Razavi, R. , Y. Maghsoudlou , M. Aalami , and M. Ghorbani . 2021. “Impact of Carboxymethyl Cellulose Coating Enriched With *Thymus Vulgaris* L. Extract on Physicochemical, Microbial, and Sensorial Properties of Fresh Hazelnut (*Corylus avellana* L.) During Storage.” Journal of Food Processing and Preservation 45: e15313.

[fsn370652-bib-0062] Razavi, R. , E. Toosi , M. Sheikholeslami , M. Konjedi , A. Hajian‐Tilaki , and A. Najafi . 2024. “Effect of *Origanum onites* L. Essential Oil and Cold Atmospheric Plasma on Physicochemical, Microbial, and Sensory Properties of Iranian White Cheese.” Journal of Food Quality 2024, no. 1: 2308789.

[fsn370652-bib-0063] Rebello, L. P. G. , A. M. Ramos , P. B. Pertuzatti , M. T. Barcia , N. Castillo‐Muñoz , and I. Hermosín‐Gutiérrez . 2014. “Flour of Banana (Musa AAA) Peel as a Source of Antioxidant Phenolic Compounds.” Food Research International 55: 397–403. 10.1016/j.foodres.2013.11.039.

[fsn370652-bib-0064] Rezvani, F. , H. Abbasi , and M. Nourani . 2020. “Effects of Protein–Polysaccharide Interactions on the Physical and Textural Characteristics of Low‐Fat Whipped Cream.” Journal of Food Processing and Preservation 44, no. 10: e14743. 10.1111/jfpp.14743.

[fsn370652-bib-0065] Riaz, A. , S. Lei , H. M. S. Akhtar , et al. 2018. “Preparation and Characterization of Chitosan‐Based Antimicrobial Active Food Packaging Film Incorporated With Apple Peel Polyphenols.” International Journal of Biological Macromolecules 114: 547–555. 10.1016/j.ijbiomac.2018.03.126. 29578019

[fsn370652-bib-0066] Rodriguez Garcia, S. L. , and V. Raghavan . 2022. “Green Extraction Techniques From Fruit and Vegetable Waste to Obtain Bioactive Compounds—A Review.” Critical Reviews in Food Science and Nutrition 62, no. 23: 6446–6466. 10.1080/10408398.2021.1901651. 33792417

[fsn370652-bib-0067] Sarabandi, K. , S. M. Jafari , A. S. Mahoonak , and A. Mohammadi . 2019. “Application of Gum Arabic and Maltodextrin for Encapsulation of Eggplant Peel Extract as a Natural Antioxidant and Color Source.” International Journal of Biological Macromolecules 140: 59–68. 31422189 10.1016/j.ijbiomac.2019.08.133

[fsn370652-bib-0068] Seo, C. W. , and B. Yoo . 2022. “Effect of Milk Protein Isolate/κ‐Carrageenan Conjugates on Rheological and Physical Properties of Whipping Cream: A Comparative Study of Maillard Conjugates and Electrostatic Complexes.” Food Science of Animal Resources 42, no. 5: 889. 10.5851/kosfa.2022.e42. 36133636 PMC9478977

[fsn370652-bib-0069] Shahidi Noghabi, M. , and M. Molaveisi . 2020. “Microencapsulation Optimization of Cinnamon Essential Oil in the Matrices of Gum Arabic, Maltodextrin, and Inulin by Spray‐Drying Using Mixture Design.” Journal of Food Process Engineering 43, no. 2: e13341.

[fsn370652-bib-0070] Shaygannia, S. , M. R. Eshaghi , M. Fazel , and M. Hashemiravan . 2021. “The Effect of Microencapsulation of Phenolic Compounds From Lemon Waste by Persian and Basil Seed Gums on the Chemical and Microbiological Properties of Mayonnaise.” Preventive Nutrition and Food Science 26, no. 1: 82. 10.3746/pnf.2021.26.1.82. 33859963 PMC8027048

[fsn370652-bib-0071] Shehzadi, K. , Q. Rubab , L. Asad , et al. 2020. “A Critical Review on Presence of Polyphenols in Commercial Varieties of Apple Peel, Their Extraction and Health Benefits.” Open Access Journal of Biogeography Science and Research 6: 18.

[fsn370652-bib-0072] Šovljanski, O. , V. Travičić , A. Tomić , et al. 2023. “From Agricultural Waste to Functional Food Products: An Overview.” Agricultural Waste: Environmental Impact, Useful Metabolites and Energy Production 31: 489–520. 10.1007/978-981-19-8774-8_18.

[fsn370652-bib-0073] Sukkwai, S. , K. Kijroongrojana , P. Chonpracha , et al. 2018. “Effects of Colorant Concentration and ‘Natural Colour’ or ‘Sodium Content’ Claim on Saltiness Perception, Consumer Liking and Emotion, and Purchase Intent of Dipping Sauces.” International Journal of Food Science & Technology 53, no. 5: 1246–1254. 10.1111/ijfs.13704.

[fsn370652-bib-0074] Tavakoli, J. , H. Abbasi , A. Zarei Jelyani , and A. Mousavi Khaneghah . 2021. “The Use of Salvia Macrosiphon and *Lepidium sativum* Linn. Seed Gums in Nanoencapsulation Processes: Improving Antioxidant Activity of Potato Skin Extract.” Journal of Food Quality 2021: 1–8.

[fsn370652-bib-0075] Taweechat, C. , T. Wongsooka , and S. Rawdkuen . 2021. “Properties of Banana (*Cavendish* spp.) Starch Film Incorporated With Banana Peel Extract and Its Application.” Molecules 26, no. 5: 1406. 10.3390/molecules26051406. 33807750 PMC7961874

[fsn370652-bib-0076] Van Nguyen, S. , and B.‐K. Lee . 2023. “Multifunctional Food Packaging Polymer Composites Based on Polyvinyl Alcohol/Cellulose Nanocrystals/Apple Peel Extract.” Cellulose 30, no. 3: 1697–1716. 10.1007/s10570-022-04976-x. 36528148

[fsn370652-bib-0077] Vieira, F. G. K. , G. D. S. C. Borges , C. Copetti , P. F. Di Pietro , E. d. C. Nunes , and R. Fett . 2011. “Phenolic Compounds and Antioxidant Activity of the Apple Flesh and Peel of Eleven Cultivars Grown in Brazil.” Scientia Horticulturae 128, no. 3: 261–266. 10.1016/j.scienta.2011.01.032.

[fsn370652-bib-0078] Vu, H. T. , C. J. Scarlett , and Q. V. Vuong . 2018. “Phenolic Compounds Within Banana Peel and Their Potential Uses: A Review.” Journal of Functional Foods 40: 238–248. 10.1016/j.jff.2017.11.006.

[fsn370652-bib-0079] Wang, Z. , X. Mei , X. Chen , et al. 2023. “Extraction and Recovery of Bioactive Soluble Phenolic Compounds From Brocade Orange ( *Citrus sinensis* ) Peels: Effect of Different Extraction Methods Thereon.” LWT 173: 114337. 10.1016/j.lwt.2022.114337.

[fsn370652-bib-0080] Wu, S. , Z. Zhang , C. Liu , and T. Ma . 2023. “Effect of pH‐Shifting and Sonication‐Assisted Treatment on Properties and Stability of Vegetable Oil‐Based Whipped Cream Stabilized by Kidney Bean Protein Aggregates.” Food Hydrocolloids 141: 108736. 10.1016/j.foodhyd.2023.108736.

[fsn370652-bib-0081] Yadav, K. , R. K. Bajaj , S. Mandal , P. Saha , and B. Mann . 2018. “Evaluation of Total Phenol Content and Antioxidant Properties of Encapsulated Grape Seed Extract in Yoghurt.” International Journal of Dairy Technology 71, no. 1: 96–104. 10.1111/1471-0307.12464.

