# Supplementary material for: The Impact of Free and Nanoencapsulated Banana and Apple Peels Extracts on the Physicochemical, Oxidative Stability, Microbial and Sensory Properties of Whipped Cream
Source: Food Sci Nutr. 2025 Jul 16;13(7):e70652. doi: 10.1002/fsn3.70652 (PMC12267888; doi:10.1002/fsn3.70652)
Supplement: Supplementary file 1 — Figure S1. [file FSN3-13-e70652-s002.zip › FigS1/FigS1.docx]

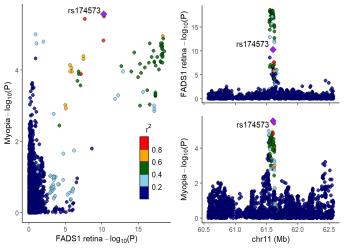

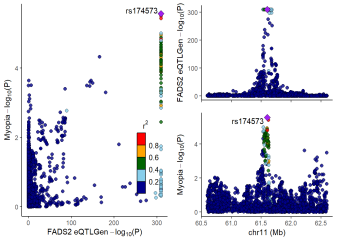


**Myopia**

**(ukb-6353)**


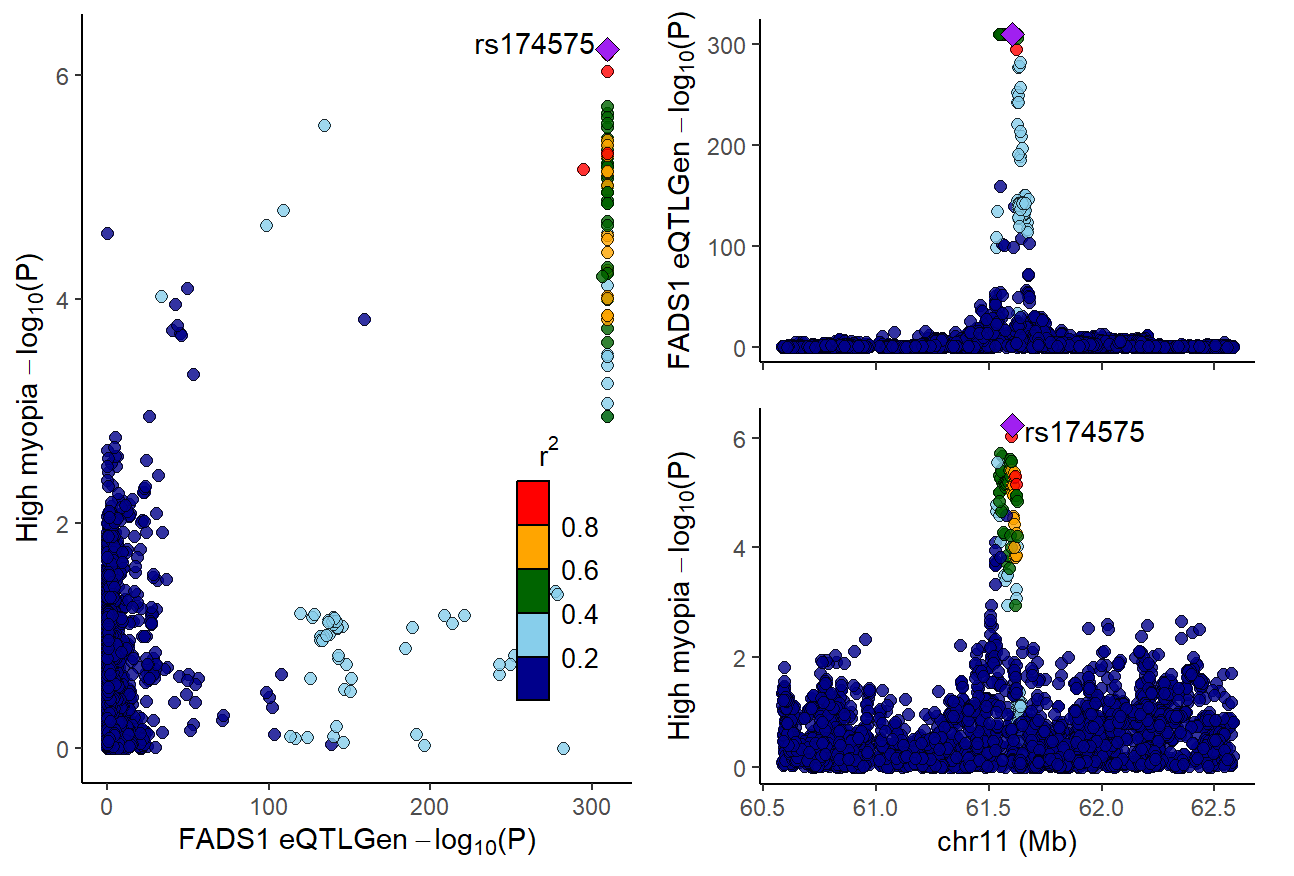

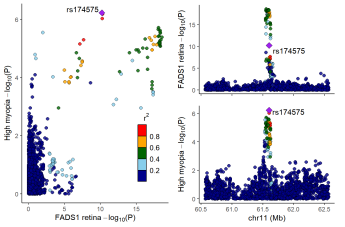

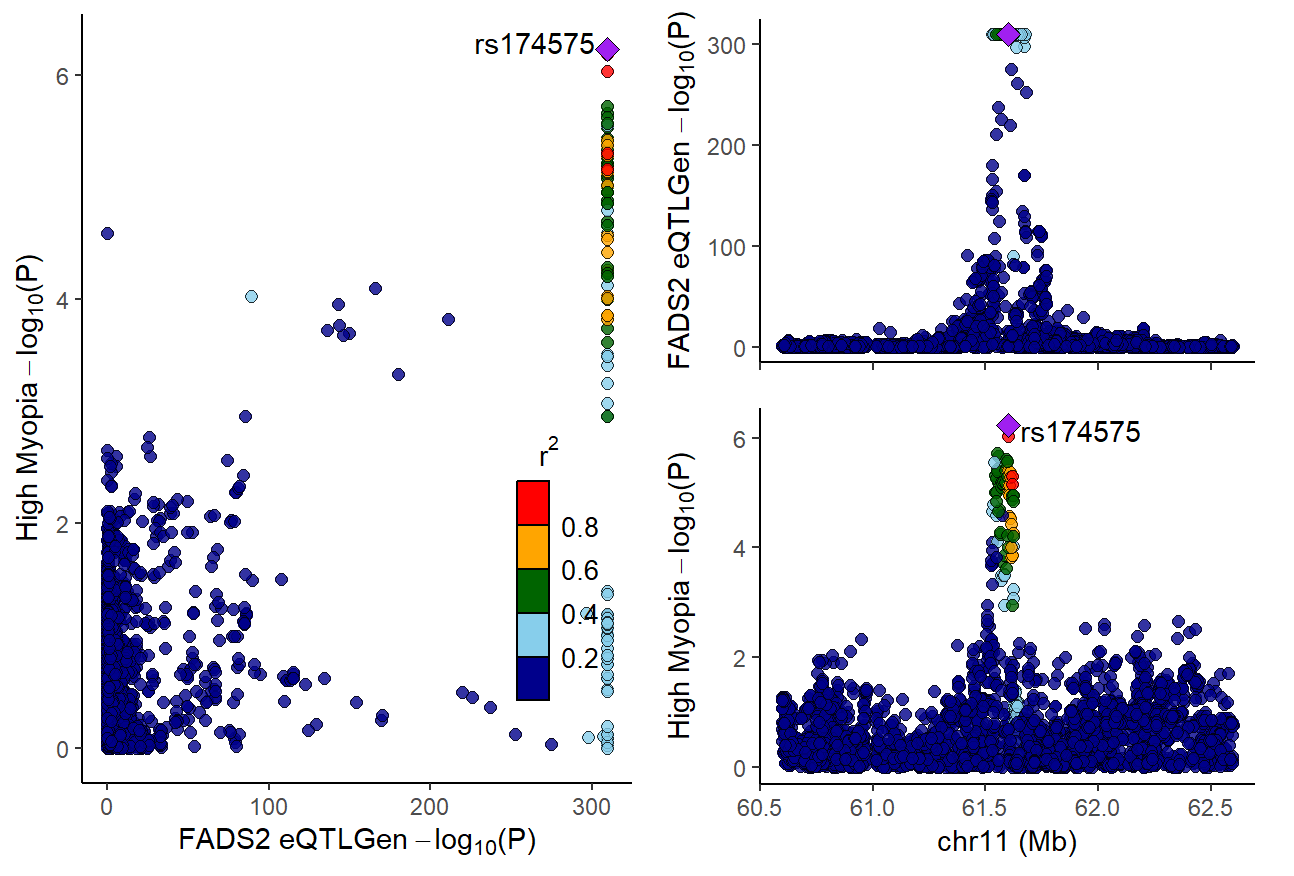


**High myopia (Boutin TS et al)**


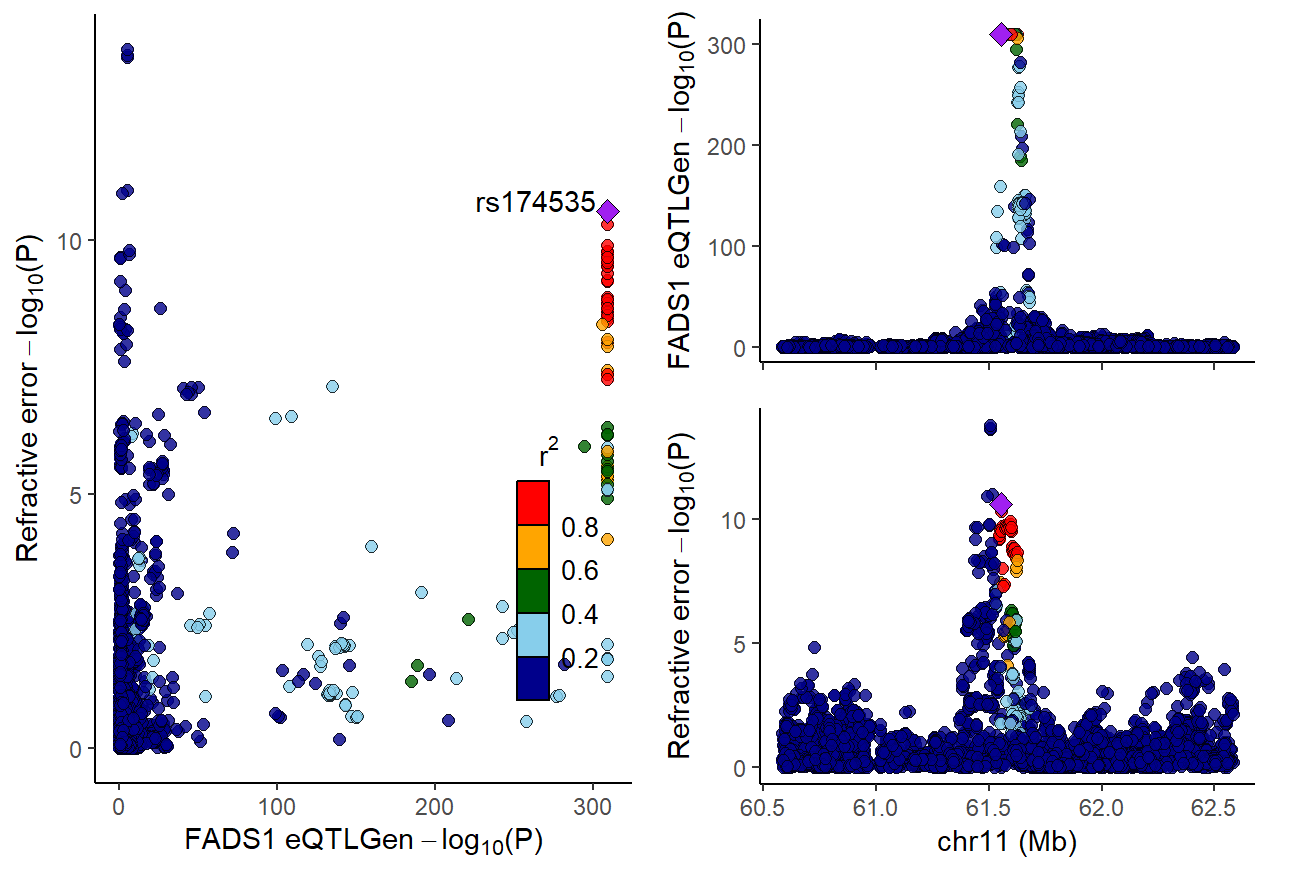

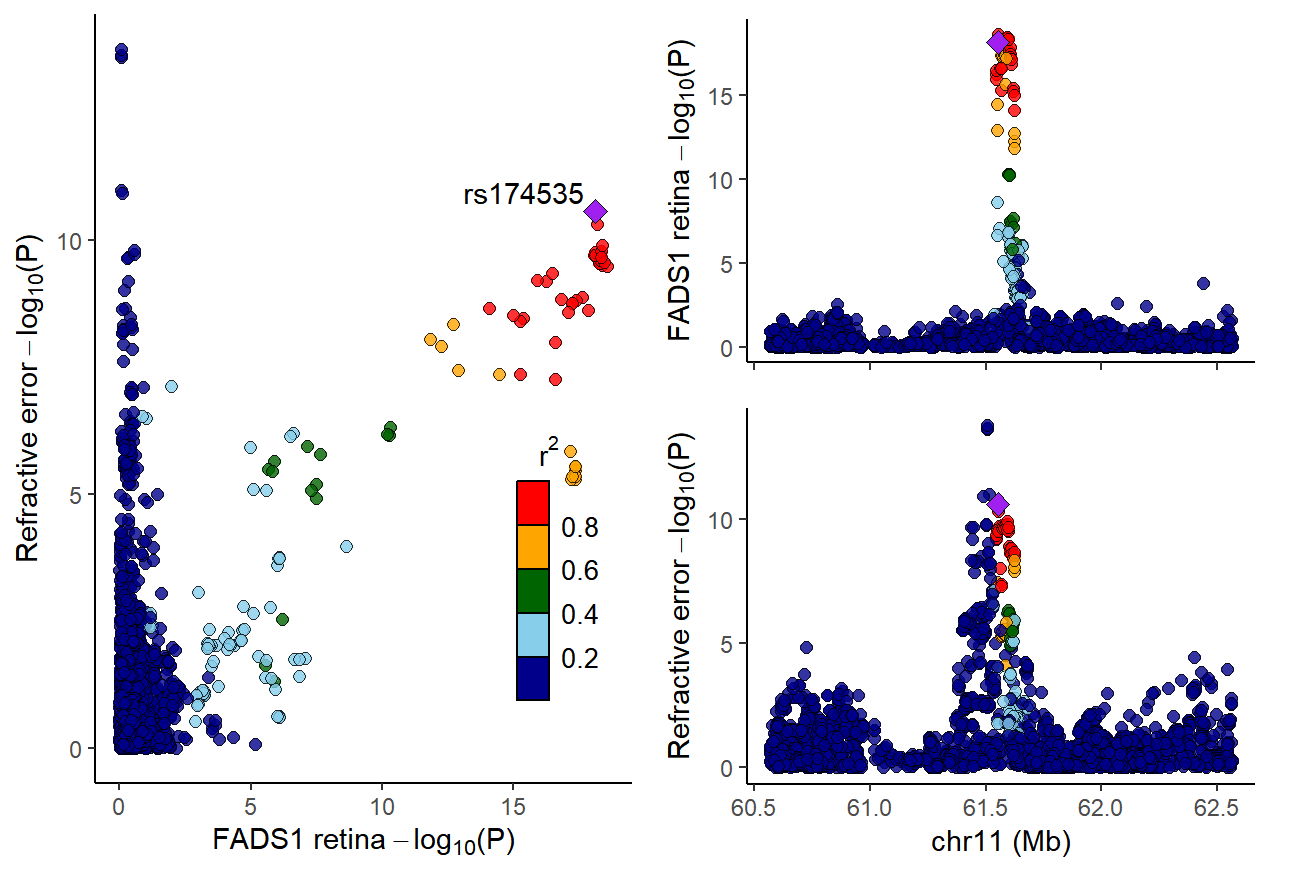

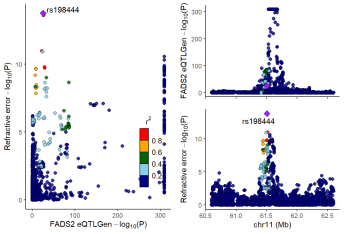


**Refractive error**

**(Hysi PG et al)**

**FADS2 from eQTLGen**

**FADS1 from eQTLGen**

**FADS1 in retina**
