# Supplementary material for: The Impact of Free and Nanoencapsulated Banana and Apple Peels Extracts on the Physicochemical, Oxidative Stability, Microbial and Sensory Properties of Whipped Cream
Source: Food Sci Nutr. 2025 Jul 16;13(7):e70652. doi: 10.1002/fsn3.70652 (PMC12267888; doi:10.1002/fsn3.70652)
Supplement: Supplementary file 2 — Table S5‐S9. [file FSN3-13-e70652-s003.zip › TableS5-S9/Table S5-S9.docx]

Table S5 Baseline characteristics of study subjects grouped by no myopia and myopia in Korean Adolescents.

|  | **No myopia (n=33)** | **Myopia (n=350)** | **P value** |
| --- | --- | --- | --- |
| **Basic information** |  |  |  |
| Sex, n(%) |  |  | 0.128 |
| men | 22 (65.8%) | 179 (51.5%) |  |
| women | 11 (34.2%) | 171 (48.5%) |  |
| Age, mean(SE), (year) | 14.94 (0.35) | 15.24 (0.10) | 0.365 |
| Education, n(%) |  |  | 0.667 |
| Primary school | 21 (53.0%) | 198 (44.1%) |  |
| Junior high school graduation | 10 (39.6%) | 133 (48.6%) |  |
| High school graduation | 2 (7.4%) | 19 (7.3%) |  |
| Height, mean(SE),(cm) | 164.04 (1.62) | 165.61 (0.58) | 0.536 |
| Weight, mean(SE),(kg) | 58.18(2.27) | 58.78 (0.85) | 0.959 |
| BMI, mean(SE),(kg/m2) | 21.49 (0.78) | 21.28 (0.24) | 0.821 |
|  |  |  |  |

| Residence, n(%) |  |  | 0.104 |
| --- | --- | --- | --- |
| Urban areas | 27 (80.6%) | 303 (90.0%) |  |
| rural areas | 6 (19.4%) | 47 (10.0%) |  |

| **Ophthalmic examination** |  |  |  |
| --- | --- | --- | --- |
| Near work time, n(%) |  |  | 0.566 |
| ＜1 | 3 (8.4%) | 16 (3.5%) |  |
| 1-2 | 11 (24.8%) | 77 (21.9%) |  |
| 3 | 4 (16.6%) | 85 (23.4%) |  |
| ＞4 | 16 (50.3%) | 172 (51.2%) |  |
| Parental myopia, n(%) |  |  | 0.391 |
| yes | 20 (56.8%) | 238 (65.8%) |  |
| no | 13 (43.2%) | 112 (34.2%) |  |
| SE, mean(SE), (D) | -0.03 (0.06) | -3.76 (0.16) | <0.001 |
|  |  |  |  |

| **Physical activity** |  |  | 0.334 |
| --- | --- | --- | --- |
| Aerobic exercise ,n(%) |  |  |  |
| Higher weekly volume* | 8 (24.1%) | 111 (32.8%) |  |
| Lower weekly volume** | 25 (75.9%) | 239 (67.2%) |  |

| **Nutrition survey** |  |  |  |
| --- | --- | --- | --- |
| Protein, mean(SE), (g/day) | 85.37 (6.92) | 78.90 (2.77) | 0.425 |
| PUFA, mean(SE) ,(g/day) | 12.92 (1.24) | 12.75 (0.51) | 0.586 |
| Zinc, mean(SE) ,(mg/day) | 11.30 (0.89) | 10.56 (0.33) | 0.322 |
| Omega-3 PUFA, mean(SE),(g/day) | 1.60 (0.18) | 1.55 (0.07) | 0.701 |
| Omega-6 PUFA, mean(SE),(g/day) | 11.29 (1.08) | 11.15 (0.45) | 0.603 |
| N-3/N-6,mean(SE) | 0.15 (0.01) | 0.15 (0.01) | 0.921 |
| Energy,mean(SE),(kcal/day) | 2,330.99 (153.04) | 2,068.88 (51.39) | 0.125 |
| EPA,median (25th, 75th), (mg/day) | 21.74 (3.49, 97.59) | 19.63 (4.75, 60.67) | 0.749 |
| DHA,median (25th,75th),(mg/day) | 103.34 (25.68, 241.31) | 60.48 (20.53, 161.51) | 0.390 |
| EPA and DHA,median (25th, 75th),(mg/day) | 103.34 (34.47, 318.54) | 83.79 (32.71, 206.64) | 0.446 |
| DHA, n(%) |  |  | 0.633 |
| T1(<10 percentile) | 2 (5.8%) | 31 (10.5%) |  |
| T2(≥10，＜90 percentile) | 26 (81.9%) | 284 (79.9%) |  |
| T3(≥90 percentile) | 5 (12.3%) | 35 (9.6%) |  |
| EPA, n(%) |  |  | 0.178 |
| T1(<10 percentile) | 4 (17.9%) | 31 (9.4%) |  |
| T2(≥10，＜90 percentile) | 23 (66.0%) | 282 (81.3%) |  |
| T3(≥90 percentile) | 6 (16.1%) | 37 (9.4%) |  |
| EPA and DHA, n(%) |  |  | 0.786 |
| T1(<10 percentile) | 3 (10.7%) | 31 (10.0%) |  |
| T2(≥10，＜90 percentile) | 25 (75.4%) | 284 (80.3%) |  |
| T3(≥90 percentile) | 5 (14.0%) | 35 (9.6%) |  |
| Omega-3 PUFA, n(%) |  |  | 0.168 |
| T1(<10 percentile) | 1 (2.9%) | 32 (11.0%) |  |
| T2(≥10，＜90 percentile) | 27 (90.2%) | 277 (78.8%) |  |
| T3(≥90 percentile) | 5(6.9%) | 41(10.2%) |  |

* Weekly physical activity limits: Moderate-intensity ≤150 min, vigorous-intensity ≤75 min, or equivalent combination (1 min vigorous = 2 min moderate).

** Weekly physical activity limits: Moderate-intensity ≥150 min, vigorous-intensity ≥75 min, or equivalent combination (1 min vigorous = 2 min moderate).

Table S6 Baseline characteristics of study subjects grouped by no high myopia and high myopia in Korean Adolescents.

|  | **No high myopia (n=326)** | **High myopia (n=57)** | **P value** |
| --- | --- | --- | --- |
| **Basic information** |  |  |  |
| Sex, n(%) |  |  | 0.527 |
| men | 172 (53.5%) | 29 (48.1%) |  |
| women | 154 (46.4%) | 28 (51.9%) |  |
| Age, mean(SE), (year) | 15.19 (0.11) | 15.35 (0.33) | 0.654 |
| Education, n(%) |  |  | 0.413 |
| Primary school | 192 (46.3%) | 27 (36.1%) |  |
| Junior high school graduation | 116 (46.2%) | 27 (57.3%) |  |
| High school graduation | 18 (7.5%) | 3 (6.6%) |  |
| Height, mean(SE),(cm) | 165.12 (0.55) | 167.50 (1.49) | 0.144 |
| Weight, mean(SE),(kg) | 57.79 (0.84) | 63.93 (2.13) | 0.003 |
| BMI, mean(SE),(kg/m^2^) | 21.05 (0.24) | 22.68 (0.63) | 0.019 |
|  |  |  |  |

| Residence, n(%) |  |  | 0.365 |
| --- | --- | --- | --- |
| Urban areas | 278 (88.7%) | 52 (92.61%) |  |
| rural areas | 48 (11.3%) | 5 (7.4%) |  |

| **Ophthalmic examination** |  |  |  |
| --- | --- | --- | --- |
| Near work time, n(%) |  |  |  |
| ＜1 |  |  |  |
| 1-2 |  |  |  |
| 3 |  |  |  |
| ＞4 |  |  |  |
| Parental myopia, n(%) |  |  |  |
| yes |  |  |  |
| no |  |  |  |
| SE, mean(SE), (D) |  |  |  |
|  |  |  |  |

| **Physical activity** |  |  | 0.611 |
| --- | --- | --- | --- |
| Aerobic exercise ,n(%) |  |  |  |
| Higher weekly volume* | 104 (32.7%) | 15 (28.8%) |  |
| Lower weekly volume** | 222 (67.3%) | 42 (71.2%) |  |

| **Nutrition survey** |  |  |  |
| --- | --- | --- | --- |
| Protein, mean(SE), (g/day) | 80.06 (2.96) | 75.94 (5.39) | 0.401 |
| PUFA, mean(SE) ,(g/day) | 12.70 (0.54) | 13.10 (1.06) | 0.706 |
| Zinc, mean(SE) ,(mg/day) | 10.75 (0.35) | 9.92 (0.67) | 0.298 |
| Omega-3 PUFA, mean(SE),(g/day) | 1.56 (0.08) | 1.54 (0.14) | 0.792 |
| Omega-6 PUFA, mean(SE),(g/day) | 11.10 (0.48) | 11.52 (0.94) | 0.679 |
| N-3/N-6,mean(SE) | 0.15 (0.01) | 0.14 (0.01) | 0.558 |
| Energy,mean(SE),(kcal/day) | 2,112.92 (55.61) | 1,963.33 (123.93) | 0.230 |
| EPA,median (25th, 75th), (mg/day) | 19.13 (4.78, 65.90) | 21.29 (3.90, 53.22) | 0.890 |
| DHA,median (25th,75th),(mg/day) | 60.83 (20.53, 167.78) | 60.48 (21.27, 133.33) | 0.727 |
| EPA and DHA,median (25th, 75th), (mg/day) | 82.73 (32.71, 227.41) | 106.26 (28.48, 177.98) | 0.871 |
| DHA, n(%) |  |  | 0.193 |
| T1(<10 percentile) | 28 (10.3%) | 5 (9.3%) |  |
| T2(≥10，＜90 percentile) | 263 (78.7%) | 48 (87.4%) |  |
| T3(≥90 percentile) | 36 (11.0%) | 4 (3.3%) |  |
| EPA, n(%) |  |  | 0.122 |
| T1(<10 percentile) | 31 (10.9%) | 4 (5.6%) |  |
| T2(≥10，＜90 percentile) | 257 (78.4%) | 48 (89.5%) |  |
| T3(≥90 percentile) | 38 (10.8%) | 5 (5.0%) |  |
| EPA and DHA, n(%) |  |  | 0.178 |
| T1(<10 percentile) | 30 (10.6%) | 4 (7.3%) |  |
| T2(≥10，＜90 percentile) | 260 (78.3%) | 49 (88.9%) |  |
| T3(≥90 percentile) | 36 (11.1%) | 4 (3.8%) |  |
| Omega-3 PUFA, n(%) |  |  | 0.877 |
| T1(<10 percentile) | 28 (10.6%) | 5 (9.1%) |  |
| T2(≥10，＜90 percentile) | 259 (79.3%) | 45 (82.1%) |  |
| T3(≥90 percentile) | 39 (10.1%) | 7 (8.9%) |  |

* Weekly physical activity limits: Moderate-intensity ≤150 min, vigorous-intensity ≤75 min, or equivalent combination (1 min vigorous = 2 min moderate).

** Weekly physical activity limits: Moderate-intensity ≥150 min, vigorous-intensity ≥75 min, or equivalent combination (1 min vigorous = 2 min moderate).

Table S7 Multiple logistic regression for the association between DHA, EPA, DHA and EPA and omega-3 PUFAs and Myopia in Korean Adolescents. Model was adjusted for age, gender, parental myopia, BMI, 24-h intake of energy, residence and aerobic exercise

|  | **Adjusted model OR (95% CI)** | **p** |
| --- | --- | --- |
| **DHA** |  |  |
| T1(<10 percentile) | 1.66(0.23,12.11) | 0.62 |
| T2(≥10，＜90 percentile) | 1.14(0.35,3.70) | 0.83 |
| T3(≥90 percentile) | Ref |  |
| **EPA** |  |  |
| T1(<10 percentile) | 0.59 (0.15, 2.35) | 0.45 |
| T2(≥10，＜90 percentile) | 1.90(0.60, 6.09) | 0.28 |
| T3(≥90 percentile) | Ref |  |
| **EPA and DHA** |  |  |
| T1(<10 percentile) | 0.86(0.11, 6.93) | 0.88 |
| T2(≥10，＜90 percentile) | 1.42 (0.46, 4.42) | 0.55 |
| T3(≥90 percentile) | Ref |  |
| **Omega-3 PUFA** |  |  |
| T1(<10 percentile) | 1.28 (0.09, 17.31) | 0.85 |
| T2(≥10，＜90 percentile) | 0.43 (0.13, 1.38) | 0.16 |
| T3(≥90 percentile) | Ref |  |

Table S8 STROBE Statement—Checklist of items that should be included in reports of ***cross-sectional studies***

|  | Item No | Recommendation | Page No |
| --- | --- | --- | --- |
| **Title and abstract** | 1 | (*a*) Indicate the study’s design with a commonly used term in the title or the abstract | 4 |
|  |  | (*b*) Provide in the abstract an informative and balanced summary of what was done and what was found | 4 |
| Introduction | | | |
| Background/rationale | 2 | Explain the scientific background and rationale for the investigation being reported | 7 |
| Objectives | 3 | State specific objectives, including any prespecified hypotheses | 8 |
| Methods | | | |
| Study design | 4 | Present key elements of study design early in the paper | 12 |
| Setting | 5 | Describe the setting, locations, and relevant dates, including periods of recruitment, exposure, follow-up, and data collection | Supplementary material  p 20-22 |
| Participants | 6 | (*a*) Give the eligibility criteria, and the sources and methods of selection of participants | Supplementary material  p 20-21 |
| Variables | 7 | Clearly define all outcomes, exposures, predictors, potential confounders, and effect modifiers. Give diagnostic criteria, if applicable | Supplementary material  P 21-22 |
| Data sources/ measurement | 8* | For each variable of interest, give sources of data and details of methods of assessment (measurement). Describe comparability of assessment methods if there is more than one group | Supplementary material  P 21-22 |
| Bias | 9 | Describe any efforts to address potential sources of bias | Supplementary material  P 22-23 |
| Study size | 10 | Explain how the study size was arrived at | Supplementary material  p 20-21 |
| Quantitative variables | 11 | Explain how quantitative variables were handled in the analyses. If applicable, describe which groupings were chosen and why | Supplementary material  P 22-23 |
| Statistical methods | 12 | (*a*) Describe all statistical methods, including those used to control for confounding | Supplementary material  P 22-23 |
|  |  | (*b*) Describe any methods used to examine subgroups and interactions | - |
|  |  | (*c*) Explain how missing data were addressed | Supplementary material  p 20-21 |
|  |  | (*d*) If applicable, describe analytical methods taking account of sampling strategy | Supplementary material  P 22-23 |
|  |  | (*e*) Describe any sensitivity analyses | Supplementary material  P 22-23 |
| Results | | | |
| Participants | 13* | (a) Report numbers of individuals at each stage of study—eg numbers potentially eligible, examined for eligibility, confirmed eligible, included in the study, completing follow-up, and analysed | 17-18;figueS2 |
|  |  | (b) Give reasons for non-participation at each stage | 18;figueS2 |
|  |  | (c) Consider use of a flow diagram | figueS2 |
| Descriptive data | 14* | (a) Give characteristics of study participants (eg demographic, clinical, social) and information on exposures and potential confounders | 18;table s6-7 |
|  |  | (b) Indicate number of participants with missing data for each variable of interest | 18;figueS2 |
| Outcome data | 15* | Report numbers of outcome events or summary measures | 18-19 |
| Main results | 16 | (*a*) Give unadjusted estimates and, if applicable, confounder-adjusted estimates and their precision (eg, 95% confidence interval). Make clear which confounders were adjusted for and why they were included | 18-19;Table 2-3 |
|  |  | (*b*) Report category boundaries when continuous variables were categorized | 18-19;Table 2-3 |
|  |  | (*c*) If relevant, consider translating estimates of relative risk into absolute risk for a meaningful time period | - |
| Other analyses | 17 | Report other analyses done—eg analyses of subgroups and interactions, and sensitivity analyses | 18-19 |
| Discussion | | | |
| Key results | 18 | Summarise key results with reference to study objectives | 21 |
| Limitations | 19 | Discuss limitations of the study, taking into account sources of potential bias or imprecision. Discuss both direction and magnitude of any potential bias | 23-24,25 |
| Interpretation | 20 | Give a cautious overall interpretation of results considering objectives, limitations, multiplicity of analyses, results from similar studies, and other relevant evidence | 23-24 |
| Generalisability | 21 | Discuss the generalisability (external validity) of the study results | 23-24 |
| Other information | | | |
| Funding | 22 | Give the source of funding and the role of the funders for the present study and, if applicable, for the original study on which the present article is based | 26 |

*Give information separately for exposed and unexposed groups.

**Note:** An Explanation and Elaboration article discusses each checklist item and gives methodological background and published examples of transparent reporting. The STROBE checklist is best used in conjunction with this article (freely available on the Web sites of PLoS Medicine at http://www.plosmedicine.org/, Annals of Internal Medicine at http://www.annals.org/, and Epidemiology at http://www.epidem.com/). Information on the STROBE Initiative is available at www.strobe-statement.org.

Table S9 STROBE-MR checklist of recommended items to address in reports of Mendelian randomization studies^1 2^

| **Item No.** | **Section** | **Checklist item** | **Relevant text from manuscript** |
| --- | --- | --- | --- |
| 1 | **TITLE and ABSTRACT** | Indicate Mendelian randomization (MR) as the study’s design in the title and/or the abstract if that is a main purpose of the study | Title page & Abstract page |
|  | **INTRODUCTION** |  |  |
| 2 | **Background** | Explain the scientific background and rationale for the reported study. What is the exposure? Is a potential causal relationship between exposure and outcome plausible? Justify why MR is a helpful method to address the study question | Introduction (paragraphs 1-4) |
| 3 | **Objectives** | State specific objectives clearly, including pre-specified causal hypotheses (if any). State that MR is a method that, under specific assumptions, intends to estimate causal effects | Introduction (paragraphs 2) & figure1 |
|  | **METHODS** |  |  |
| 4 | **Study design and data sources** | Present key elements of the study design early in the article. Consider including a table listing sources of data for all phases of the study. For each data source contributing to the analysis, describe the following: |  |
|  | a) | Setting: Describe the study design and the underlying population, if possible. Describe the setting, locations, and relevant dates, including periods of recruitment, exposure, follow-up, and data collection, when available. | Methods & Supplementary Methods (GWAS Summary Statistics for Myopia phenotype; GWAS Summary Statistics for omega-3 PUFAs) |
|  | b) | Participants: Give the eligibility criteria, and the sources and methods of selection of participants. Report the sample size, and whether any power or sample size calculations were carried out prior to the main analysis | Methods & Supplementary Methods (GWAS Summary Statistics for Myopia phenotype; GWAS Summary Statistics for omega-3 PUFAs) |
|  | c) | Describe measurement, quality control and selection of genetic variants | Methods & Supplementary Methods (GWAS Summary Statistics for Myopia phenotype; GWAS Summary Statistics for omega-3 PUFAs) |
|  | d) | For each exposure, outcome, and other relevant variables, describe methods of assessment and diagnostic criteria for diseases | Methods & Supplementary Methods (GWAS Summary Statistics for Myopia phenotype; GWAS Summary Statistics for omega-3 PUFAs) |
|  | e) | Provide details of ethics committee approval and participant informed consent, if relevant | Methods (Paragraph 1) |
| 5 | **Assumptions** | Explicitly state the three core IV assumptions for the main analysis (relevance, independence and exclusion restriction) as well assumptions for any additional or sensitivity analysis | Methods & Supplementary Methods (Two-sample MR ) |
| 6 | **Statistical methods: main analysis** | Describe statistical methods and statistics used |  |
|  | a) | Describe how quantitative variables were handled in the analyses (i.e., scale, units, model) | Table S1 |
|  | b) | Describe how genetic variants were handled in the analyses and, if applicable, how their weights were selected | Methods & Supplementary Methods (Selection of fatty acid instrumental variables (IVs)) |
|  | c) | Describe the MR estimator (e.g. two-stage least squares, Wald ratio) and related statistics. Detail the included covariates and, in case of two-sample MR, whether the same covariate set was used for adjustment in the two samples | Methods & Supplementary Methods ( Two-sample MR ) |
|  | d) | Explain how missing data were addressed | N/A |
|  | e) | If applicable, indicate how multiple testing was addressed | Methods & Supplementary Methods (Two-sample MR;SMR and colocalization analysis ) |
| 7 | **Assessment of assumptions** | Describe any methods or prior knowledge used to assess the assumptions or justify their validity | Methods & Supplementary Methods (Two-sample MR;SMR and colocalization analysis ) |
| 8 | **Sensitivity analyses and additional analyses** | Describe any sensitivity analyses or additional analyses performed (e.g. comparison of effect estimates from different approaches, independent replication, bias analytic techniques, validation of instruments, simulations) | Methods & Supplementary Methods (Two-sample MR;SMR and colocalization analysis ) |
| 9 | **Software and pre-registration** |  |  |
|  | a) | Name statistical software and package(s), including version and settings used | Methods & Supplementary Methods (Two-sample MR ) |
|  | b) | State whether the study protocol and details were pre-registered (as well as when and where) | N/A |
|  | **RESULTS** |  |  |
| 10 | **Descriptive data** |  |  |
|  | a) | Report the numbers of individuals at each stage of included studies and reasons for exclusion. Consider use of a flow diagram | Table S1 |
|  | b) | Report summary statistics for phenotypic exposure(s), outcome(s), and other relevant variables (e.g. means, SDs, proportions) | Table S1 |
|  | c) | If the data sources include meta-analyses of previous studies, provide the assessments of heterogeneity across these studies | N/A |
|  | d) | For two-sample MR:  i.  Provide justification of the similarity of the genetic variant-exposure associations between the exposure and outcome samples  ii.  Provide information on the number of individuals who overlap between the exposure and outcome studies | N/A |
| 11 | **Main results** |  |  |
|  | a) | Report the associations between genetic variant and exposure, and between genetic variant and outcome, preferably on an interpretable scale | Results(Two-Sample MR analysis) ; Figure 2 |
|  | b) | Report MR estimates of the relationship between exposure and outcome, and the measures of uncertainty from the MR analysis, on an interpretable scale, such as odds ratio or relative risk per SD difference | Results(Two-Sample MR analysis) ; Figure 2 |
|  | c) | If relevant, consider translating estimates of relative risk into absolute risk for a meaningful time period | NR |
|  | d) | Consider plots to visualize results (e.g. forest plot, scatterplot of associations between genetic variants and outcome versus between genetic variants and exposure) | Figure 2 |
| 12 | **Assessment of assumptions** |  |  |
|  | a) | Report the assessment of the validity of the assumptions | Figure 2 |
|  | b) | Report any additional statistics (e.g., assessments of heterogeneity across genetic variants, such as *I^2^*, Q statistic or E-value) | Figure 2 |
| 13 | **Sensitivity analyses and additional analyses** |  |  |
|  | a) | Report any sensitivity analyses to assess the robustness of the main results to violations of the assumptions | TableS4;Figure 2 |
|  | b) | Report results from other sensitivity analyses or additional analyses | TableS4;Figure 2 |
|  | c) | Report any assessment of direction of causal relationship (e.g., bidirectional MR) | Results(SMR and colocalization analysis) |
|  | d) | When relevant, report and compare with estimates from non-MR analyses | Results(SMR and colocalization analysis) |
|  | e) | Consider additional plots to visualize results (e.g., leave-one-out analyses) | FigureS1 |
|  | **DISCUSSION** |  |  |
| 14 | **Key results** | Summarize key results with reference to study objectives | Discussion (paragraph 1) |
| 15 | **Limitations** | Discuss limitations of the study, taking into account the validity of the IV assumptions, other sources of potential bias, and imprecision. Discuss both direction and magnitude of any potential bias and any efforts to address them | Discussion (Strengths and weaknesses) |
| 16 | **Interpretation** |  |  |
|  | a) | Meaning: Give a cautious overall interpretation of results in the context of their limitations and in comparison with other studies | Discussion (Strengths and weaknesses) |
|  | b) | Mechanism: Discuss underlying biological mechanisms that could drive a potential causal relationship between the investigated exposure and the outcome, and whether the gene-environment equivalence assumption is reasonable. Use causal language carefully, clarifying that IV estimates may provide causal effects only under certain assumptions | Discussion (Potential mechanisms of omega-3 PUFAs in suppressing myopia through effects on the choroid) |
|  | c) | Clinical relevance: Discuss whether the results have clinical or public policy relevance, and to what extent they inform effect sizes of possible interventions | Conclusion |
| 17 | **Generalizability** | Discuss the generalizability of the study results (a) to other populations, (b) across other exposure periods/timings, and (c) across other levels of exposure | Conclusion |
|  | **OTHER INFORMATION** |  |  |
| 18 | **Funding** | Describe sources of funding and the role of funders in the present study and, if applicable, sources of funding for the databases and original study or studies on which the present study is based | Funding |
| 19 | **Data and data sharing** | Provide the data used to perform all analyses or report where and how the data can be accessed, and reference these sources in the article. Provide the statistical code needed to reproduce the results in the article, or report whether the code is publicly accessible and if so, where | Data availability |
| 20 | **Conflicts of Interest** | All authors should declare all potential conflicts of interest | Conflict of interest |

This checklist is copyrighted by the Equator Network under the Creative Commons Attribution 3.0 Unported (CC BY 3.0) license.

1. Skrivankova VW, Richmond RC, Woolf BAR, Yarmolinsky J, Davies NM, Swanson SA, et al. Strengthening the Reporting of Observational Studies in Epidemiology using Mendelian Randomization (STROBE-MR) Statement. JAMA. 2021;under review.

2. Skrivankova VW, Richmond RC, Woolf BAR, Davies NM, Swanson SA, VanderWeele TJ, et al. Strengthening the Reporting of Observational Studies in Epidemiology using Mendelian Randomisation (STROBE-MR): Explanation and Elaboration. BMJ. 2021;375:n2233.
