# Supplementary material for: The Impact of Free and Nanoencapsulated Banana and Apple Peels Extracts on the Physicochemical, Oxidative Stability, Microbial and Sensory Properties of Whipped Cream
Source: Food Sci Nutr. 2025 Jul 16;13(7):e70652. doi: 10.1002/fsn3.70652 (PMC12267888; doi:10.1002/fsn3.70652)
Supplement: Supplementary file 3 — Data S1. [file FSN3-13-e70652-s001.zip › Supplementary materials/Supplementary materials.docx]

**Supplementary Table**

Table S5 Baseline characteristics of study subjects grouped by no myopia and myopia in Korean Adolescents.

|  | **No myopia (n=33)** | **Myopia (n=350)** | **P value** |
| --- | --- | --- | --- |
| **Basic information** |  |  |  |
| Sex, n(%) |  |  | 0.128 |
| men | 22 (65.8%) | 179 (51.5%) |  |
| women | 11 (34.2%) | 171 (48.5%) |  |
| Age, mean(SE), (year) | 14.94 (0.35) | 15.24 (0.10) | 0.365 |
| Education, n(%) |  |  | 0.667 |
| Primary school | 21 (53.0%) | 198 (44.1%) |  |
| Junior high school graduation | 10 (39.6%) | 133 (48.6%) |  |
| High school graduation | 2 (7.4%) | 19 (7.3%) |  |
| Height, mean(SE),(cm) | 164.04 (1.62) | 165.61 (0.58) | 0.536 |
| Weight, mean(SE),(kg) | 58.18(2.27) | 58.78 (0.85) | 0.959 |
| BMI, mean(SE),(kg/m2) | 21.49 (0.78) | 21.28 (0.24) | 0.821 |
|  |  |  |  |

| Residence, n(%) |  |  | 0.104 |
| --- | --- | --- | --- |
| Urban areas | 27 (80.6%) | 303 (90.0%) |  |
| rural areas | 6 (19.4%) | 47 (10.0%) |  |

| **Ophthalmic examination** |  |  |  |
| --- | --- | --- | --- |
| Near work time, n(%) |  |  | 0.566 |
| ＜1 | 3 (8.4%) | 16 (3.5%) |  |
| 1-2 | 11 (24.8%) | 77 (21.9%) |  |
| 3 | 4 (16.6%) | 85 (23.4%) |  |
| ＞4 | 16 (50.3%) | 172 (51.2%) |  |
| Parental myopia, n(%) |  |  | 0.391 |
| yes | 20 (56.8%) | 238 (65.8%) |  |
| no | 13 (43.2%) | 112 (34.2%) |  |
| SE, mean(SE), (D) | -0.03 (0.06) | -3.76 (0.16) | <0.001 |
|  |  |  |  |

| **Physical activity** |  |  | 0.334 |
| --- | --- | --- | --- |
| Aerobic exercise ,n(%) |  |  |  |
| Higher weekly volume* | 8 (24.1%) | 111 (32.8%) |  |
| Lower weekly volume** | 25 (75.9%) | 239 (67.2%) |  |

| **Nutrition survey** |  |  |  |
| --- | --- | --- | --- |
| Protein, mean(SE), (g/day) | 85.37 (6.92) | 78.90 (2.77) | 0.425 |
| PUFA, mean(SE) ,(g/day) | 12.92 (1.24) | 12.75 (0.51) | 0.586 |
| Zinc, mean(SE) ,(mg/day) | 11.30 (0.89) | 10.56 (0.33) | 0.322 |
| Omega-3 PUFA, mean(SE),(g/day) | 1.60 (0.18) | 1.55 (0.07) | 0.701 |
| Omega-6 PUFA, mean(SE),(g/day) | 11.29 (1.08) | 11.15 (0.45) | 0.603 |
| N-3/N-6,mean(SE) | 0.15 (0.01) | 0.15 (0.01) | 0.921 |
| Energy,mean(SE),(kcal/day) | 2,330.99 (153.04) | 2,068.88 (51.39) | 0.125 |
| EPA,median (25th, 75th), (mg/day) | 21.74 (3.49, 97.59) | 19.63 (4.75, 60.67) | 0.749 |
| DHA,median (25th,75th),(mg/day) | 103.34 (25.68, 241.31) | 60.48 (20.53, 161.51) | 0.390 |
| EPA and DHA,median (25th, 75th),(mg/day) | 103.34 (34.47, 318.54) | 83.79 (32.71, 206.64) | 0.446 |
| DHA, n(%) |  |  | 0.633 |
| T1(<10 percentile) | 2 (5.8%) | 31 (10.5%) |  |
| T2(≥10，＜90 percentile) | 26 (81.9%) | 284 (79.9%) |  |
| T3(≥90 percentile) | 5 (12.3%) | 35 (9.6%) |  |
| EPA, n(%) |  |  | 0.178 |
| T1(<10 percentile) | 4 (17.9%) | 31 (9.4%) |  |
| T2(≥10，＜90 percentile) | 23 (66.0%) | 282 (81.3%) |  |
| T3(≥90 percentile) | 6 (16.1%) | 37 (9.4%) |  |
| EPA and DHA, n(%) |  |  | 0.786 |
| T1(<10 percentile) | 3 (10.7%) | 31 (10.0%) |  |
| T2(≥10，＜90 percentile) | 25 (75.4%) | 284 (80.3%) |  |
| T3(≥90 percentile) | 5 (14.0%) | 35 (9.6%) |  |
| Omega-3 PUFA, n(%) |  |  | 0.168 |
| T1(<10 percentile) | 1 (2.9%) | 32 (11.0%) |  |
| T2(≥10，＜90 percentile) | 27 (90.2%) | 277 (78.8%) |  |
| T3(≥90 percentile) | 5(6.9%) | 41(10.2%) |  |

* Weekly physical activity limits: Moderate-intensity ≤150 min, vigorous-intensity ≤75 min, or equivalent combination (1 min vigorous = 2 min moderate).

** Weekly physical activity limits: Moderate-intensity ≥150 min, vigorous-intensity ≥75 min, or equivalent combination (1 min vigorous = 2 min moderate).

Table S6 Baseline characteristics of study subjects grouped by no high myopia and high myopia in Korean Adolescents.

|  | **No high myopia (n=326)** | **High myopia (n=57)** | **P value** |
| --- | --- | --- | --- |
| **Basic information** |  |  |  |
| Sex, n(%) |  |  | 0.527 |
| men | 172 (53.5%) | 29 (48.1%) |  |
| women | 154 (46.4%) | 28 (51.9%) |  |
| Age, mean(SE), (year) | 15.19 (0.11) | 15.35 (0.33) | 0.654 |
| Education, n(%) |  |  | 0.413 |
| Primary school | 192 (46.3%) | 27 (36.1%) |  |
| Junior high school graduation | 116 (46.2%) | 27 (57.3%) |  |
| High school graduation | 18 (7.5%) | 3 (6.6%) |  |
| Height, mean(SE),(cm) | 165.12 (0.55) | 167.50 (1.49) | 0.144 |
| Weight, mean(SE),(kg) | 57.79 (0.84) | 63.93 (2.13) | 0.003 |
| BMI, mean(SE),(kg/m^2^) | 21.05 (0.24) | 22.68 (0.63) | 0.019 |
|  |  |  |  |

| Residence, n(%) |  |  | 0.365 |
| --- | --- | --- | --- |
| Urban areas | 278 (88.7%) | 52 (92.61%) |  |
| rural areas | 48 (11.3%) | 5 (7.4%) |  |

| **Ophthalmic examination** |  |  |  |
| --- | --- | --- | --- |
| Near work time, n(%) |  |  |  |
| ＜1 |  |  |  |
| 1-2 |  |  |  |
| 3 |  |  |  |
| ＞4 |  |  |  |
| Parental myopia, n(%) |  |  |  |
| yes |  |  |  |
| no |  |  |  |
| SE, mean(SE), (D) |  |  |  |
|  |  |  |  |

| **Physical activity** |  |  | 0.611 |
| --- | --- | --- | --- |
| Aerobic exercise ,n(%) |  |  |  |
| Higher weekly volume* | 104 (32.7%) | 15 (28.8%) |  |
| Lower weekly volume** | 222 (67.3%) | 42 (71.2%) |  |

| **Nutrition survey** |  |  |  |
| --- | --- | --- | --- |
| Protein, mean(SE), (g/day) | 80.06 (2.96) | 75.94 (5.39) | 0.401 |
| PUFA, mean(SE) ,(g/day) | 12.70 (0.54) | 13.10 (1.06) | 0.706 |
| Zinc, mean(SE) ,(mg/day) | 10.75 (0.35) | 9.92 (0.67) | 0.298 |
| Omega-3 PUFA, mean(SE),(g/day) | 1.56 (0.08) | 1.54 (0.14) | 0.792 |
| Omega-6 PUFA, mean(SE),(g/day) | 11.10 (0.48) | 11.52 (0.94) | 0.679 |
| N-3/N-6,mean(SE) | 0.15 (0.01) | 0.14 (0.01) | 0.558 |
| Energy,mean(SE),(kcal/day) | 2,112.92 (55.61) | 1,963.33 (123.93) | 0.230 |
| EPA,median (25th, 75th), (mg/day) | 19.13 (4.78, 65.90) | 21.29 (3.90, 53.22) | 0.890 |
| DHA,median (25th,75th),(mg/day) | 60.83 (20.53, 167.78) | 60.48 (21.27, 133.33) | 0.727 |
| EPA and DHA,median (25th, 75th), (mg/day) | 82.73 (32.71, 227.41) | 106.26 (28.48, 177.98) | 0.871 |
| DHA, n(%) |  |  | 0.193 |
| T1(<10 percentile) | 28 (10.3%) | 5 (9.3%) |  |
| T2(≥10，＜90 percentile) | 263 (78.7%) | 48 (87.4%) |  |
| T3(≥90 percentile) | 36 (11.0%) | 4 (3.3%) |  |
| EPA, n(%) |  |  | 0.122 |
| T1(<10 percentile) | 31 (10.9%) | 4 (5.6%) |  |
| T2(≥10，＜90 percentile) | 257 (78.4%) | 48 (89.5%) |  |
| T3(≥90 percentile) | 38 (10.8%) | 5 (5.0%) |  |
| EPA and DHA, n(%) |  |  | 0.178 |
| T1(<10 percentile) | 30 (10.6%) | 4 (7.3%) |  |
| T2(≥10，＜90 percentile) | 260 (78.3%) | 49 (88.9%) |  |
| T3(≥90 percentile) | 36 (11.1%) | 4 (3.8%) |  |
| Omega-3 PUFA, n(%) |  |  | 0.877 |
| T1(<10 percentile) | 28 (10.6%) | 5 (9.1%) |  |
| T2(≥10，＜90 percentile) | 259 (79.3%) | 45 (82.1%) |  |
| T3(≥90 percentile) | 39 (10.1%) | 7 (8.9%) |  |

* Weekly physical activity limits: Moderate-intensity ≤150 min, vigorous-intensity ≤75 min, or equivalent combination (1 min vigorous = 2 min moderate).

** Weekly physical activity limits: Moderate-intensity ≥150 min, vigorous-intensity ≥75 min, or equivalent combination (1 min vigorous = 2 min moderate).

Table S7 Multiple logistic regression for the association between DHA, EPA, DHA and EPA and omega-3 PUFAs and Myopia in Korean Adolescents. Model was adjusted for age, gender, parental myopia, BMI, 24-h intake of energy, residence and aerobic exercise

|  | **Adjusted model OR (95% CI)** | **p** |
| --- | --- | --- |
| **DHA** |  |  |
| T1(<10 percentile) | 1.66(0.23,12.11) | 0.62 |
| T2(≥10，＜90 percentile) | 1.14(0.35,3.70) | 0.83 |
| T3(≥90 percentile) | Ref |  |
| **EPA** |  |  |
| T1(<10 percentile) | 0.59 (0.15, 2.35) | 0.45 |
| T2(≥10，＜90 percentile) | 1.90(0.60, 6.09) | 0.28 |
| T3(≥90 percentile) | Ref |  |
| **EPA and DHA** |  |  |
| T1(<10 percentile) | 0.86(0.11, 6.93) | 0.88 |
| T2(≥10，＜90 percentile) | 1.42 (0.46, 4.42) | 0.55 |
| T3(≥90 percentile) | Ref |  |
| **Omega-3 PUFA** |  |  |
| T1(<10 percentile) | 1.28 (0.09, 17.31) | 0.85 |
| T2(≥10，＜90 percentile) | 0.43 (0.13, 1.38) | 0.16 |
| T3(≥90 percentile) | Ref |  |

Table S8 STROBE Statement—Checklist of items that should be included in reports of ***cross-sectional studies***

|  | Item No | Recommendation | Page No |
| --- | --- | --- | --- |
| **Title and abstract** | 1 | (*a*) Indicate the study’s design with a commonly used term in the title or the abstract | 4 |
|  |  | (*b*) Provide in the abstract an informative and balanced summary of what was done and what was found | 4 |
| Introduction | | | |
| Background/rationale | 2 | Explain the scientific background and rationale for the investigation being reported | 7 |
| Objectives | 3 | State specific objectives, including any prespecified hypotheses | 8 |
| Methods | | | |
| Study design | 4 | Present key elements of study design early in the paper | 12 |
| Setting | 5 | Describe the setting, locations, and relevant dates, including periods of recruitment, exposure, follow-up, and data collection | Supplementary material  p 20-22 |
| Participants | 6 | (*a*) Give the eligibility criteria, and the sources and methods of selection of participants | Supplementary material  p 20-21 |
| Variables | 7 | Clearly define all outcomes, exposures, predictors, potential confounders, and effect modifiers. Give diagnostic criteria, if applicable | Supplementary material  P 21-22 |
| Data sources/ measurement | 8* | For each variable of interest, give sources of data and details of methods of assessment (measurement). Describe comparability of assessment methods if there is more than one group | Supplementary material  P 21-22 |
| Bias | 9 | Describe any efforts to address potential sources of bias | Supplementary material  P 22-23 |
| Study size | 10 | Explain how the study size was arrived at | Supplementary material  p 20-21 |
| Quantitative variables | 11 | Explain how quantitative variables were handled in the analyses. If applicable, describe which groupings were chosen and why | Supplementary material  P 22-23 |
| Statistical methods | 12 | (*a*) Describe all statistical methods, including those used to control for confounding | Supplementary material  P 22-23 |
|  |  | (*b*) Describe any methods used to examine subgroups and interactions | - |
|  |  | (*c*) Explain how missing data were addressed | Supplementary material  p 20-21 |
|  |  | (*d*) If applicable, describe analytical methods taking account of sampling strategy | Supplementary material  P 22-23 |
|  |  | (*e*) Describe any sensitivity analyses | Supplementary material  P 22-23 |
| Results | | | |
| Participants | 13* | (a) Report numbers of individuals at each stage of study—eg numbers potentially eligible, examined for eligibility, confirmed eligible, included in the study, completing follow-up, and analysed | 17-18;figueS2 |
|  |  | (b) Give reasons for non-participation at each stage | 18;figueS2 |
|  |  | (c) Consider use of a flow diagram | figueS2 |
| Descriptive data | 14* | (a) Give characteristics of study participants (eg demographic, clinical, social) and information on exposures and potential confounders | 18;table s6-7 |
|  |  | (b) Indicate number of participants with missing data for each variable of interest | 18;figueS2 |
| Outcome data | 15* | Report numbers of outcome events or summary measures | 18-19 |
| Main results | 16 | (*a*) Give unadjusted estimates and, if applicable, confounder-adjusted estimates and their precision (eg, 95% confidence interval). Make clear which confounders were adjusted for and why they were included | 18-19;Table 2-3 |
|  |  | (*b*) Report category boundaries when continuous variables were categorized | 18-19;Table 2-3 |
|  |  | (*c*) If relevant, consider translating estimates of relative risk into absolute risk for a meaningful time period | - |
| Other analyses | 17 | Report other analyses done—eg analyses of subgroups and interactions, and sensitivity analyses | 18-19 |
| Discussion | | | |
| Key results | 18 | Summarise key results with reference to study objectives | 21 |
| Limitations | 19 | Discuss limitations of the study, taking into account sources of potential bias or imprecision. Discuss both direction and magnitude of any potential bias | 23-24,25 |
| Interpretation | 20 | Give a cautious overall interpretation of results considering objectives, limitations, multiplicity of analyses, results from similar studies, and other relevant evidence | 23-24 |
| Generalisability | 21 | Discuss the generalisability (external validity) of the study results | 23-24 |
| Other information | | | |
| Funding | 22 | Give the source of funding and the role of the funders for the present study and, if applicable, for the original study on which the present article is based | 26 |

*Give information separately for exposed and unexposed groups.

**Note:** An Explanation and Elaboration article discusses each checklist item and gives methodological background and published examples of transparent reporting. The STROBE checklist is best used in conjunction with this article (freely available on the Web sites of PLoS Medicine at http://www.plosmedicine.org/, Annals of Internal Medicine at http://www.annals.org/, and Epidemiology at http://www.epidem.com/). Information on the STROBE Initiative is available at www.strobe-statement.org.

Table S9 STROBE-MR checklist of recommended items to address in reports of Mendelian randomization studies^1 2^

| **Item No.** | **Section** | **Checklist item** | **Relevant text from manuscript** |
| --- | --- | --- | --- |
| 1 | **TITLE and ABSTRACT** | Indicate Mendelian randomization (MR) as the study’s design in the title and/or the abstract if that is a main purpose of the study | Title page & Abstract page |
|  | **INTRODUCTION** |  |  |
| 2 | **Background** | Explain the scientific background and rationale for the reported study. What is the exposure? Is a potential causal relationship between exposure and outcome plausible? Justify why MR is a helpful method to address the study question | Introduction (paragraphs 1-4) |
| 3 | **Objectives** | State specific objectives clearly, including pre-specified causal hypotheses (if any). State that MR is a method that, under specific assumptions, intends to estimate causal effects | Introduction (paragraphs 2) & figure1 |
|  | **METHODS** |  |  |
| 4 | **Study design and data sources** | Present key elements of the study design early in the article. Consider including a table listing sources of data for all phases of the study. For each data source contributing to the analysis, describe the following: |  |
|  | a) | Setting: Describe the study design and the underlying population, if possible. Describe the setting, locations, and relevant dates, including periods of recruitment, exposure, follow-up, and data collection, when available. | Methods & Supplementary Methods (GWAS Summary Statistics for Myopia phenotype; GWAS Summary Statistics for omega-3 PUFAs) |
|  | b) | Participants: Give the eligibility criteria, and the sources and methods of selection of participants. Report the sample size, and whether any power or sample size calculations were carried out prior to the main analysis | Methods & Supplementary Methods (GWAS Summary Statistics for Myopia phenotype; GWAS Summary Statistics for omega-3 PUFAs) |
|  | c) | Describe measurement, quality control and selection of genetic variants | Methods & Supplementary Methods (GWAS Summary Statistics for Myopia phenotype; GWAS Summary Statistics for omega-3 PUFAs) |
|  | d) | For each exposure, outcome, and other relevant variables, describe methods of assessment and diagnostic criteria for diseases | Methods & Supplementary Methods (GWAS Summary Statistics for Myopia phenotype; GWAS Summary Statistics for omega-3 PUFAs) |
|  | e) | Provide details of ethics committee approval and participant informed consent, if relevant | Methods (Paragraph 1) |
| 5 | **Assumptions** | Explicitly state the three core IV assumptions for the main analysis (relevance, independence and exclusion restriction) as well assumptions for any additional or sensitivity analysis | Methods & Supplementary Methods (Two-sample MR ) |
| 6 | **Statistical methods: main analysis** | Describe statistical methods and statistics used |  |
|  | a) | Describe how quantitative variables were handled in the analyses (i.e., scale, units, model) | Table S1 |
|  | b) | Describe how genetic variants were handled in the analyses and, if applicable, how their weights were selected | Methods & Supplementary Methods (Selection of fatty acid instrumental variables (IVs)) |
|  | c) | Describe the MR estimator (e.g. two-stage least squares, Wald ratio) and related statistics. Detail the included covariates and, in case of two-sample MR, whether the same covariate set was used for adjustment in the two samples | Methods & Supplementary Methods ( Two-sample MR ) |
|  | d) | Explain how missing data were addressed | N/A |
|  | e) | If applicable, indicate how multiple testing was addressed | Methods & Supplementary Methods (Two-sample MR;SMR and colocalization analysis ) |
| 7 | **Assessment of assumptions** | Describe any methods or prior knowledge used to assess the assumptions or justify their validity | Methods & Supplementary Methods (Two-sample MR;SMR and colocalization analysis ) |
| 8 | **Sensitivity analyses and additional analyses** | Describe any sensitivity analyses or additional analyses performed (e.g. comparison of effect estimates from different approaches, independent replication, bias analytic techniques, validation of instruments, simulations) | Methods & Supplementary Methods (Two-sample MR;SMR and colocalization analysis ) |
| 9 | **Software and pre-registration** |  |  |
|  | a) | Name statistical software and package(s), including version and settings used | Methods & Supplementary Methods (Two-sample MR ) |
|  | b) | State whether the study protocol and details were pre-registered (as well as when and where) | N/A |
|  | **RESULTS** |  |  |
| 10 | **Descriptive data** |  |  |
|  | a) | Report the numbers of individuals at each stage of included studies and reasons for exclusion. Consider use of a flow diagram | Table S1 |
|  | b) | Report summary statistics for phenotypic exposure(s), outcome(s), and other relevant variables (e.g. means, SDs, proportions) | Table S1 |
|  | c) | If the data sources include meta-analyses of previous studies, provide the assessments of heterogeneity across these studies | N/A |
|  | d) | For two-sample MR:  i.  Provide justification of the similarity of the genetic variant-exposure associations between the exposure and outcome samples  ii.  Provide information on the number of individuals who overlap between the exposure and outcome studies | N/A |
| 11 | **Main results** |  |  |
|  | a) | Report the associations between genetic variant and exposure, and between genetic variant and outcome, preferably on an interpretable scale | Results(Two-Sample MR analysis) ; Figure 2 |
|  | b) | Report MR estimates of the relationship between exposure and outcome, and the measures of uncertainty from the MR analysis, on an interpretable scale, such as odds ratio or relative risk per SD difference | Results(Two-Sample MR analysis) ; Figure 2 |
|  | c) | If relevant, consider translating estimates of relative risk into absolute risk for a meaningful time period | NR |
|  | d) | Consider plots to visualize results (e.g. forest plot, scatterplot of associations between genetic variants and outcome versus between genetic variants and exposure) | Figure 2 |
| 12 | **Assessment of assumptions** |  |  |
|  | a) | Report the assessment of the validity of the assumptions | Figure 2 |
|  | b) | Report any additional statistics (e.g., assessments of heterogeneity across genetic variants, such as *I^2^*, Q statistic or E-value) | Figure 2 |
| 13 | **Sensitivity analyses and additional analyses** |  |  |
|  | a) | Report any sensitivity analyses to assess the robustness of the main results to violations of the assumptions | TableS4;Figure 2 |
|  | b) | Report results from other sensitivity analyses or additional analyses | TableS4;Figure 2 |
|  | c) | Report any assessment of direction of causal relationship (e.g., bidirectional MR) | Results(SMR and colocalization analysis) |
|  | d) | When relevant, report and compare with estimates from non-MR analyses | Results(SMR and colocalization analysis) |
|  | e) | Consider additional plots to visualize results (e.g., leave-one-out analyses) | FigureS1 |
|  | **DISCUSSION** |  |  |
| 14 | **Key results** | Summarize key results with reference to study objectives | Discussion (paragraph 1) |
| 15 | **Limitations** | Discuss limitations of the study, taking into account the validity of the IV assumptions, other sources of potential bias, and imprecision. Discuss both direction and magnitude of any potential bias and any efforts to address them | Discussion (Strengths and weaknesses) |
| 16 | **Interpretation** |  |  |
|  | a) | Meaning: Give a cautious overall interpretation of results in the context of their limitations and in comparison with other studies | Discussion (Strengths and weaknesses) |
|  | b) | Mechanism: Discuss underlying biological mechanisms that could drive a potential causal relationship between the investigated exposure and the outcome, and whether the gene-environment equivalence assumption is reasonable. Use causal language carefully, clarifying that IV estimates may provide causal effects only under certain assumptions | Discussion (Potential mechanisms of omega-3 PUFAs in suppressing myopia through effects on the choroid) |
|  | c) | Clinical relevance: Discuss whether the results have clinical or public policy relevance, and to what extent they inform effect sizes of possible interventions | Conclusion |
| 17 | **Generalizability** | Discuss the generalizability of the study results (a) to other populations, (b) across other exposure periods/timings, and (c) across other levels of exposure | Conclusion |
|  | **OTHER INFORMATION** |  |  |
| 18 | **Funding** | Describe sources of funding and the role of funders in the present study and, if applicable, sources of funding for the databases and original study or studies on which the present study is based | Funding |
| 19 | **Data and data sharing** | Provide the data used to perform all analyses or report where and how the data can be accessed, and reference these sources in the article. Provide the statistical code needed to reproduce the results in the article, or report whether the code is publicly accessible and if so, where | Data availability |
| 20 | **Conflicts of Interest** | All authors should declare all potential conflicts of interest | Conflict of interest |

This checklist is copyrighted by the Equator Network under the Creative Commons Attribution 3.0 Unported (CC BY 3.0) license.

1. Skrivankova VW, Richmond RC, Woolf BAR, Yarmolinsky J, Davies NM, Swanson SA, et al. Strengthening the Reporting of Observational Studies in Epidemiology using Mendelian Randomization (STROBE-MR) Statement. JAMA. 2021;under review.

2. Skrivankova VW, Richmond RC, Woolf BAR, Davies NM, Swanson SA, VanderWeele TJ, et al. Strengthening the Reporting of Observational Studies in Epidemiology using Mendelian Randomisation (STROBE-MR): Explanation and Elaboration. BMJ. 2021;375:n2233.

**Supplementary Figure**


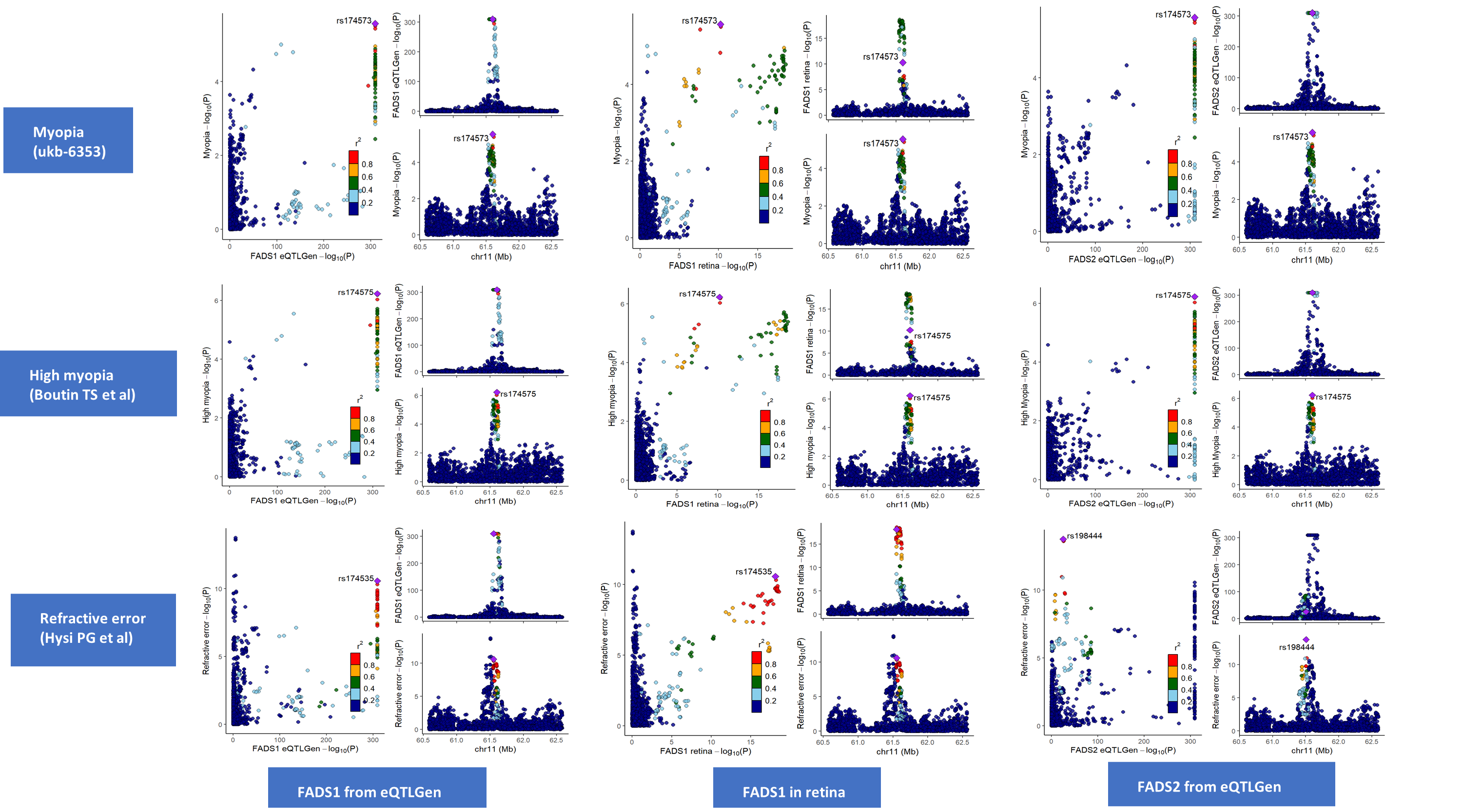


Figure S1 Visualization of colocalization analysis of FASD1 and FADS2 gene from both blood and retinal tissue and three traits of myopia.

**Supplementary Methods**.

The study was divided into two parts: 1) To investigate causal relationship between omega-3 PUFAs and myopia traits, a two-sample Mendelian randomization (MR) study was conducted using publicly accessible genome-wide association study (GWAS) data in European populations. Plasma level of total omega-3 PUFAs, DHA, EPA, Docosapentaenoic acid (DPA) and Alpha-Linolenic Acid (ALA) were set as exposures, and myopia, RSE and high myopia were set as outcomes. To assess possible interactions between the FADS1 and FADS2 gene expressions and the three myopia phenotypes, summary data-based Mendelian randomization (SMR) and colocalization analysis were used (**Figure1**). 2) Due to the absence of publicly available GWAS data on myopia based on large sample sizes of East Asian populations, we conducted multivariable regression analysis using data from the KNHANES database to investigate the relationship between omega-3 PUFAs and spherical equivalent (SE) and high myopia. The original GWAS protocols and data collection methods received approval from the ethics committee, and written informed consent was obtained from each participant prior to data collection.

**GWAS Summary Statistics for Myopia phenotype**

All three traits of myopia were used as outcome in two-sample MR study, including myopia, RSE and high myopia. Myopia data were downloaded from MRC Integrative Epidemiology Unit (IEU) OpenGWAS database (ukb-b-6353; 37,362 cases, 423,174 controls)^[1]^. From 2006 to 2010, baseline questionnaires, measurements, and biological samples were collected at 22 assessment centers throughout the UK. Myopia was defined as “glasses/contact lenses: For shortsightedness” in the myopia data in UK Biobank.

RSE data was downloaded from the GWAS catalog (a meta-analysis conducted by Hysi et al^[2]^, N=351,091). The RSE meta-analysis initially comprised 542,934 individuals of European ancestry, making it the largest published GWAS datasets for refractive error, encompassing SE data in diopters from five distinct studies: two subsets of participants from the UK Biobank (UKB 1 and UKB 2), the Genetic Epidemiology Research on Adult Health and Aging (GERA) cohorts, the 23andMe personal genomics company customer base, and the Consortium for Refractive Error and Myopia (CREAM) Study. UKB1 represents spherical equivalent data derived from directly measured sphere and cylinder power in the UK Biobank. UKB2 is a dataset obtained using a support vector machine (SVM) model to predict participants' spherical equivalent based on other parameters. In the CREAM study, refractive errors in participants aged 25 and older were measured through refractometry. The participants of 23andMe are volunteers from the personal genomics company^[2]^. GERA is part of the Kaiser Permanente Research Program on Genes, Environment, and Health (RPGEH), which has been detailed elsewhere, including 34,998 adults (aged 25 and above) who self-identified as non-Hispanic white and had at least one spherical equivalent assessment between 2008 and 2014^[3]^. Due to restricted access to 23andMe data in GWAS catalog, GWAS Summary Statistics for refractive error in our study do not include 23andMe customer base, resulting in N=351,091 participants in the GWAS Summary Statistics. β coefficients and standard error were estimated by z score, minor allele frequency (MAF) and sample size, according to these following equations .$\beta=\frac{Z}{\sqrt{{2\times MAF(1-MAF)}^{2}{(Z}^{2}+N)}} SE=\frac{1}{\sqrt{{2\times MAF(1-MAF)}^{2}{(Z}^{2}+N)}}$

High myopia data were also downloaded from the GWAS catalog (N=2737 cases, 47635 controls). For high myopia data, 50372 UK Biobank participants of British ancestry were used to obtain summary statistics in this GWAS of high myopia ^[4]^. In this study, high myopia cases were characterized by an average SE of less than -6 diopters in both eyes, and controls were individuals with average SE no less than -0.5 diopters.

Since both myopia and high myopia GWAS were derived exclusively from UK Biobank participants, while the spherical equivalent GWAS was primarily (though not exclusively) drawn from UK Biobank populations. Thereby minimizing population heterogeneity across these endpoint measures.

**GWAS Summary Statistics for omega-3 PUFAs**

To avoid the potential bias by sample overlap, we used the publicly accessible GWAS datasets for plasma levels of total omega-3 PUFAs, DHA, DPA, EPA and ALA from non-UK Biobank participants from European descent, which has the great proportion of variation in that fatty acid. Previous study revealed that while the Heart and Aging Research in Genomic Epidemiology (CHARGE) consortium database provided genetic instruments explaining greater phenotypic variance for ALA, EPA and DPA, the METSIM/FINRISK datasets demonstrated superior explanatory power for DHA variation, with total omega-3 levels exclusively available in the Metabolic Syndrome in Men (METSIM) and National FINRISK studies^[5]^. We consequently integrated both databases to enhance result robustness.

GWAS Data from METSIM/FINRISK were used for total omega-3 PUFAs (N = 8751 cases) and DHA (N = 8751 cases). METSIM study enrolled 10,197 male participants aged 45-73 years, systematically recruited from Kuopio's population registry in Eastern Finland between 2005-2010, while researchers selected 10,192 individuals from three northern provinces (North Karelia, Oulu, and Lapland) who participated in survey between 1992 and 2007 in the FINRISK study. Both cohorts underwent exome sequencing and clinical measurements of cardiovascular and metabolic health indicators^[5]^, with nuclear magnetic resonance (NMR) spectroscopy employed for quantifying fatty acids of varying chain lengths^[6, 7]^ .

Data from the Cohorts for CHARGE consortium were used for DPA (N=8866 cases) , EPA (N=8866 cases) and ALA (N=8866 cases). The CHARGE study utilized gas chromatography to separate fatty acids and included a meta-analysis of genome-wide association studies (GWAS) from European descent from across 5 GWAS^[8]^.

Since using Finnish populations (METSIM/FINRISK) as the exposure and UK Biobank (UKB) populations as the outcome may introduce population stratification bias, we employed DHA data (N=8,866 cases) from the CHARGE consortium as a replication dataset to validate the METSIM/FINRISK-derived DHA results.

The details of the above GWAS Summary Statistics were described in **Table S1**.

**Selection of fatty acid instrumental variables (IVs)**

Referencing a previously published MR study^[9]^, IVs were extracted from GWAS of total omega-3 PUFAs, DHA, DPA, EPA and ALA, respectively. Patchen etal defined IVs with linkage disequilibrium r2 < 0.01 on the basis of distinct genetic loci identified in published GWAS studies on omega-3 PUFAs. These IVs explained a relatively high variance and were fewer in number, which avoid potential horizontal pleiotropy. IVs with F-statistics less than 10 were removed. The IVs for DHA from the CHARGE consortium were selected based on the DHA-associated genetic variants identified in the METSIM/FINRISK study.

The details of IVs in our study are listed in **Table S2**.

For total omega-3 PUFAs and DHA, we used 6 and 5 SNPs that explained 6.3% and 7.4% of the variance. For DPA, EPA and ALA, we used 2, 3 and 1 SNPs that explained 8.5%, 2.2% and 1.4% of the variance. For each instrument variable, all F statistics exceeded 10.

The FADS region, which encode key fatty acid desaturase enzymes, has been biologically proven to play a role in omega-3 PUFAs metabolism^[10, 11]^. The FADS1 (chromosome 11: 61567099–61584475, GRCh37) and FADS2 genes (chromosome 11: 61583675–61634826, GRCh37) encode D5D and D6D enzymes, respectively, which catalyze rate-limiting desaturase reactions in the biosynthesis of omega-3 PUFAs ^[10]^. To identify whether the results were driven by SNP within the FADS region and to reduce susceptibility to horizontal pleiotropy bias^[12]^, secondary analysis was conducted at the protein level by only an IV within the FADS region. Since rs174546 has been identified from the results of CHARGE as an instrument for fatty acid desaturase activity and represents the FADS gene cluster in previous studies ^[10, 11]^, we used LDlink (https://ldlink.nci.nih.gov/) to calculate the linkage disequilibrium (LD) between rs174546 and IVs located on chromosome 11. We then selected an IV with the maximum r2, indicating the highest LD, to represent FADS region. Details were showed in **Table S3**.

**Two-sample MR**

The MR design is based on three core assumptions^[13]^. Firstly, the genetic instruments should have a strong association with the biomarker under investigation. Secondly, these genetic instruments must influence the outcome solely through the exposure and not through an alternative biological pathway that is unrelated to the exposure. Lastly, the genetic instruments must remain unassociated with any confounders that could affect the relationship between the exposure and the outcome. The MR analysis utilized the R statistical software along with the TwoSampleMR package (version 0.5.6). For SNP in palindromic sequences, if the minor allele frequency is greater than 0.42, the SNP was considered uninformative and would be excluded when harmonization. Inverse variance weighted (IVW) method was used as principal analysis, followed by MR-Egger, weighted-median, simple mode and weighted mode methods. Only IVW or Wald ratio were used for 2 IVs or 1 IV when MR was conducted. MR-Robust Adjusted Profile Score (MR-RAPS) was further conducted to accommodates weak instruments and reduces pleiotropy. An association was identified as P value less than 0.01 (0.05/5, Bonferroni correction). For sensitivity analysis, Egger intercept was calculated to evaluate horizontal pleiotropy and heterogeneity test was also conducted.

**Expression quantitative trait locus (eQTL) Data**

We used cis-eQTLs to represent the effect of 2 genes (FADS1 and FADS2) on omega-3 PUFAs. The all summary cis-eQTLs data for gene expression of 2 genes in both blood and retina were used. The blood data was downloaded from the eQTLGen Consortium (https://www.eqtlgen.org/)^[14]^ and the retina data was downloaded from the Institute of Human Genetics at the University of Regensburg (<https://www-huge.uni-regensburg.de/databases.html>) ^[15]^. In this mega-analysis of eQTLs, gene expression regulation of healthy retinal tissue (n = 311) was investigated by Strunz et al and largest available retinal cis-eQTL data set at that time was established, where 403,151 significant eQTL variants that regulate 3007 genes were identified^[15]^. The SNPs within 1mb upstream and downstream of FASD1 and FADS2 gene loci were included for analysis.

**SMR and colocalization analysis**

We utilized SMR tool to identify associations between gene expression level of FADS1 and FADS2 and complex traits of myopia, RSE and high myopia using summary data from eQTLs and GWAS studies. 1000 Genomes European reference was used to calculate LD. The Heterogeneity in dependent instruments (HEIDI) test method was also performed using 20 SNPs. The criteria for supporting potential association was defined as those that (1) passed SMR P value with Bonferroni correction< 0.05/4. (2) P value of HEIDI test > 0.05. SMR & HEIDI methods and software tool were download from Yang Lab^[16]^.

Colocalization analysis was conducted to evaluate the potential interactions between gene expression and traits of myopia. It is a method to assess whether there is evidence for a shared causal variant in the genomic region associated with exposure and outcome. The analysis was conducted utilizing the coloc R package, and PPH4 > 0.8 was defined as strong evidence of colocalization while 0.8> PPH4 > 0.5 was defined as moderate evidence of colocalization. Additionally, the probability of [PPH4/(PPH3 + PPH4)] was defined as the probability of colocalization conditional on the presence of a causal variant for outcomes^[17, 18]^. Full cis-eQTLs of two genes were included for analysis.

**Data Source and Participants from KNHANES**

The KNHANES is a nationwide population-based cross-sectional survey by the Division of Chronic Disease Surveillance, Korea Centers for Disease Control and Prevention, including health interview, nutrition survey, and health examination. Survey by complex, multi-stage probability sampling design was conducted every three years from 1998 to 2005, and became an annual survey plan in 2007^[19]^.

We obtained data from the seventh KNHANES survey (KNHANES VII, 2016-2018) to estimate the association between omega-3 PUFAs and two phenotype of myopia (myopia, RSE and high myopia). The design of the KNHANES VII study adhered to the principles outlined in the Declaration of Helsinki. All participants involved in the survey provided their consent by signing an informed consent form. Further information regarding the study design and methodologies are provided elsewhere^[20]^. Participants whose characteristic lacked results of ophthalmic examination, 24-h dietary recall and covariates were excluded from our study.

**Assessment of refraction**

In mobile examination centers, participants underwent noncycloplegic autorefraction by Topcon KR8800 autorefractor (Topcon, Tokyo, Japan) three times in both eyes. Relaxation of accommodation was stimulated with a picture target under standard background lighting conditions by autorefractor. The spherical equivalent (diopter) is calculated by adding the sum of the sphere power with half of the cylinder power. Myopia and high myopia are defined as spherical equivalent (SE) less than or equal to -0.50D and -6.0D .

**Assessment of N-3 PUFAs**

24-h dietary recall method was used to assess dietary intake, which can provide insights into the recent dietary consumption patterns of participants. Professionally trained dietitians interviewed the participants to recall and detail all food products consumed during the preceding day. Dietary intakes including total PUFAs, total omega-3 PUFAs (g/day), total omega-6 PUFAs (g/day), EPA (mg/day), DHA (mg/day), protein (g/day) and daily energy intake (kcal/day) were calculated in KNHANES.

**Assessment of covariates**

To control for confounding factors except for basic variables and nutritional variables, we included education, parental myopia, physical activity and ‘urban/rural’ variables which met two criteria: (1) supported by at least moderate-strength evidence in the International Myopia Institute (IMI) risk factor guidelines^[21]^, and (2) available in the KNHANES database. Demographic variables including age, sex ,education and residence (urban/rural), and anthropometric variables including height and weight which were measured to the nearest 0.1 cm and 0.1 kg were defined as basic information for individuals. Body mass index (BMI) was determined by weight (kg) divided by height squared (m2). Variables about physical activity include weekly aerobic exercise time. Other ophthalmic survey include near work time (h/day) and parental myopia. Age, BMI, height, weight, refraction and dietary intakes were defined as continuous variables, and sex, education, parental myopia and near work time were defined as categorical variables.

**Statistical analysis**

Statistical analysis was performed with the R software (version 4.3.1) and complex sampling design and sampling weights of KNHANES were taken into account by the complex samples packages “survey”. P value was two-sided and less than 0.05 was regraded as statistical significance. Participants were divided into two groups based on their myopia status: a myopia group and a non-myopia group, as well as a high myopia group and a non-high myopia group. Mean (standard error) was used for continuous variables corresponding to normal distribution, and median (25th percentile, 75th percentile) was used for continuous variables corresponding to non-normal distribution. For categorical variables, number and percentages were used. T-tests or wilcoxon rank sum tests were used for analysis of continuous variables, and chis quared test was used for analysis of categorical variables. Dietary intakes of total omega-3 PUFAs, DHA plus EPA, DHA and EPA were further divided into three parts as follows: Tertile 1 (< 10th percentile), Tertile 2 (≥ 10th to 90th percentile), and Tertile 3 (> 90th percentile). Multiple logistic and linear regression was performed to evaluate the association between omega-3 PUFAs and myopia. Model was adjusted for age, gender, parental myopia, BMI,24-h intake of energy and residence and aerobic exercise .

**Reference**

[1] Li F. F. et al. Causal Relationships Between Glycemic Traits and Myopia. Invest Ophthalmol Vis Sci. 2023; **64**(3): 7.

[2] Hysi P. G. et al. Meta-analysis of 542,934 subjects of European ancestry identifies new genes and mechanisms predisposing to refractive error and myopia. Nat Genet. 2020; **52**(4): 401-407.

[3] Banda Y. et al. Characterizing Race/Ethnicity and Genetic Ancestry for 100,000 Subjects in the Genetic Epidemiology Research on Adult Health and Aging (GERA) Cohort. Genetics. 2015; **200**(4): 1285-95.

[4] Boutin T. S. et al. Insights into the genetic basis of retinal detachment. Hum Mol Genet. 2020; **29**(4): 689-702.

[5] Locke A. E. et al. Exome sequencing of Finnish isolates enhances rare-variant association power. Nature. 2019; **572**(7769): 323-328.

[6] Laakso M. et al. The Metabolic Syndrome in Men study: a resource for studies of metabolic and cardiovascular diseases. J Lipid Res. 2017; **58**(3): 481-493.

[7] Borodulin K. et al. Cohort Profile: The National FINRISK Study. Int J Epidemiol. 2018; **47**(3): 696-696i.

[8] Lemaitre R. N. et al. Genetic loci associated with plasma phospholipid n-3 fatty acids: a meta-analysis of genome-wide association studies from the CHARGE Consortium. PLoS Genet. 2011; **7**(7): e1002193.

[9] Patchen B. K. et al. Investigating Associations of Omega-3 Fatty Acids, Lung Function Decline, and Airway Obstruction. Am J Respir Crit Care Med. 2023; **208**(8): 846-857.

[10] Haycock P. C. et al. The association between genetically elevated polyunsaturated fatty acids and risk of cancer. EBioMedicine. 2023; **91**: 104510.

[11] Jones H. J. et al. Associations between plasma fatty acid concentrations and schizophrenia: a two-sample Mendelian randomisation study. Lancet Psychiatry. 2021; **8**(12): 1062-1070.

[12] Holmes M. V. et al. Integrating genomics with biomarkers and therapeutic targets to invigorate cardiovascular drug development. Nat Rev Cardiol. 2021; **18**(6): 435-453.

[13] Lawlor D. A. et al. Mendelian randomization: using genes as instruments for making causal inferences in epidemiology. Stat Med. 2008; **27**(8): 1133-63.

[14] Võsa U. et al. Large-scale cis- and trans-eQTL analyses identify thousands of genetic loci and polygenic scores that regulate blood gene expression. Nat Genet. 2021; **53**(9): 1300-1310.

[15] Strunz T. et al. A mega-analysis of expression quantitative trait loci in retinal tissue. PLoS Genet. 2020; **16**(9): e1008934.

[16] Zhu Z. et al. Integration of summary data from GWAS and eQTL studies predicts complex trait gene targets. Nat Genet. 2016; **48**(5): 481-7.

[17] Wang K. et al. Evaluating the impact of glucokinase activation on risk of cardiovascular disease: a Mendelian randomisation analysis. Cardiovasc Diabetol. 2022; **21**(1): 192.

[18] Bi Y. et al. Lipids, lipid-modifying drug target genes and migraine: a Mendelian randomization study. J Headache Pain. 2023; **24**(1): 112.

[19] Kweon S. et al. Data resource profile: the Korea National Health and Nutrition Examination Survey (KNHANES). Int J Epidemiol. 2014; **43**(1): 69-77.

[20] Park H. A. The Korea national health and nutrition examination survey as a primary data source. Korean J Fam Med. 2013; **34**(2): 79.

[21] Morgan I. G. et al. IMI Risk Factors for Myopia. Invest Ophthalmol Vis Sci. 2021; **62**(5): 3.
